# Predictive Tool Use and Willingness for Surgery in Patients With Knee Osteoarthritis: A Randomized Clinical Trial

**DOI:** 10.1001/jamanetworkopen.2024.0890

**Published:** 2024-03-08

**Authors:** Yushy Zhou, Lauren Patten, Tim Spelman, Samantha Bunzli, Peter F. M. Choong, Michelle M. Dowsey, Chris Schilling

**Affiliations:** 1 Department of Surgery, The University of Melbourne, Melbourne, Victoria, Australia; 2 Department of Orthopaedic Surgery, St Vincent’s Hospital, Melbourne, Victoria, Australia; 3 School of Health Sciences and Social Work, Griffith University, Nathan Campus, Brisbane, Queensland, Australia; 4 Physiotherapy Department, Royal Brisbane and Women’s Hospital, Brisbane, Queensland, Australia

## Abstract

**Importance:**

Despite the increasing number of tools available to predict the outcomes of total knee arthroplasty (TKA), the effect of these predictive tools on patient decision-making remains uncertain.

**Objective:**

To assess the effect of an online predictive tool on patient-reported willingness to undergo TKA.

**Design, Setting, and Participants:**

This parallel, double-masked, 2-arm randomized clinical trial compared predictive tool use with treatment as usual (TAU). The study was conducted between June 30, 2022, and July 31, 2023. Participants were followed up for 6 months after enrollment. Participants were recruited from a major Australian private health insurance company and from the surgical waiting list for publicly funded TKA at a tertiary hospital. Eligible participants had unilateral knee osteoarthritis, were contemplating TKA, and had previously tried nonsurgical interventions, such as lifestyle modifications, physiotherapy, and pain medications.

**Intervention:**

The intervention group was provided access to an online predictive tool at the beginning of the study. This tool offered information regarding the likelihood of improvement in quality of life if patients chose to undergo TKA. The predictions were based on the patient’s age, sex, and baseline symptoms. Conversely, the control group received TAU without access to the predictive tool.

**Main Outcomes and Measures:**

The primary outcome measure was the reduction in participants’ willingness to undergo surgery at 6 months after tool use as measured by binomial logistic regression. Secondary outcome measures included participant treatment preference and the quality of their decision-making process as measured by the Knee Decision Quality Instrument.

**Results:**

Of 211 randomized participants (mean [SD] age, 65.8 [8.3] years; 118 female [55.9%]), 105 were allocated to the predictive tool group and 106 to the TAU group. After adjusting for baseline differences in willingness for surgery, the predictive tool did not significantly reduce the primary outcome of willingness for surgery at 6 months (adjusted odds ratio, 0.85; 95% CI, 0.42-1.71; *P* = .64).

**Conclusions and Relevance:**

Despite the absence of treatment effect on willingness for TKA, predictive tools might still enhance health outcomes of patients with knee osteoarthritis. Additional research is needed to optimize the design and implementation of predictive tools, address limitations, and fully understand their effect on the decision-making process in TKA.

**Trial Registration:**

ANZCTR.org.au Identifier: ACTRN12622000072718

## Key Points

**Question:**

What are the effects of predictive tool use on willingness for surgery in patients with knee osteoarthritis considering total knee arthroplasty?

**Findings:**

In this randomized clinical trial of 211 participants assigned to predictive tool use vs treatment as usual, predictive tool use did not significantly change patient willingness for surgery at 6 months.

**Meaning:**

These findings indicate that additional research is needed to optimize patient decision-making in total knee arthroplasty.

## Teaser

This randomized clinical trial assesses the effect of an online predictive tool on patient-reported willingness to undergo total knee arthroplasty.

## Introduction

Rapid advancements in artificial intelligence technologies have led to the development of numerous clinical predictive tools, including those for patients with knee osteoarthritis (OA) considering total knee arthroplasty (TKA).^1,2,3,4,5^ While the integration of predictive tools into clinical practice is progressing quickly, few of these tools have undergone rigorous evaluation through randomized clinical trials (RCTs).^6,7^ Consequently, the effectiveness of these tools in surgical decision-making for both patients and clinicians remains uncertain.

At the population level, studies have reported that approximately 10% to 20% of patients are dissatisfied after undergoing TKA.^8,9^ The risk of such outcomes is, however, not evenly distributed among patients.^10^ As such, predictive tools may provide patients with individualized information about surgical outcomes to assist in their decision-making process.^11^ In turn, this individualized information may result in patients who may not currently benefit from surgery pursuing nonsurgical interventions, such as lifestyle changes and physiotherapy, which have been shown to be effective at improving pain, function, and quality of life.^12,13,14,15^ The breadth of care and treatment pathways for knee OA highlights the importance of predictive tools in providing patients with an opportunity for shared decision-making.^16,17^

The SMART Choice (Knee) tool is one such predictive tool developed using the St Vincent’s Hospital Arthroplasty Outcomes (SMART) Registry.^18^ This tool aims to predict patient improvement in health-related quality of life after primary TKA. Despite the development of tools like SMART Choice, the scarcity of clinical trials assessing their effect on patient decision-making is evident.^6,7^ To address this research gap, we conducted an RCT to evaluate the effect of the SMART Choice tool on patient willingness to undergo TKA.

## Methods

### Study Registration and Ethical Approval

This parallel, double-masked, 2-arm RCT was prospectively registered with the Australian and New Zealand Clinical Trials Registry (ACTRN12622000072718). The study was conducted between June 30, 2022, and July 31, 2023. Participants were followed up for a period of 6 months post enrollment. Ethical approval was obtained from the human research ethics committees at St Vincent’s Hospital, Melbourne and The University of Melbourne. The study protocol was published prospectively in an open access journal prior to commencement of the RCT (Supplement 1).^19^ Written informed consent was obtained from all potential participants prior to enrollment. This study adheres to the Consolidated Standards of Reporting Trials (CONSORT) guideline for RCTs.^20^

### Study Design and Participants

The study was coordinated from a single university research center affiliated with a tertiary public hospital. Recruitment was open from June 30, 2022, to January 31, 2023, in line with study protocol timelines. Participants were recruited from 2 sites: St Vincent’s Hospital in Melbourne (SVHM) and the national membership base of the Hospitals Contribution Fund (HCF), the third largest private health insurance company in Australia. Participants from the SVHM site were patients with knee OA on the waiting list for TKA who had already completed consultation with an orthopedic surgeon. Participants from the HCF were recruited via an online newsletter that was available to all paying HCF members in Australia. Those who felt they were potentially suitable for the study submitted an expression of interest and underwent eligibility screening. Eligible participants were then asked to complete a baseline questionnaire (which included variables required to generate a prediction), and those randomized to the predictive tool group received a predicted outcome report, whereas those randomized to the treatment-as-usual (TAU) group did not receive the report. Total follow-up for the study was 6 months after enrollment. Participants were eligible for the study if they were aged 45 years or older, had unilateral knee OA, were considering a primary unilateral TKA, had tried nonsurgical treatments (eg, lifestyle changes, physiotherapy, or pain relief medication), understood English, and had access to a computer with internet.

### Interventions

The intervention group used the SMART Choice tool, which is used to predict improvement in health-related quality of life after TKA using data from the SMART Registry.^21^ The tool was developed using both machine learning and logistic regression techniques. Further details about the tool have been published elsewhere.^18^ Participants accessed the tool through the study website at the time of enrollment, providing baseline information to receive a predicted likelihood for improvement (intervention group only) reported as deciles ranging from 1 to 10 (eMethods 3 in Supplement 2). Lower deciles indicated a lower likelihood for improvement. The control group received TAU. The TAU group inputted the same variables as the intervention group (including predictors needed for the tool) to the study website but did not have access to the predictive tool or its reported predictions.^22^ Further information about the tool, including how the predictions are reported, can be found in eMethods 3 in Supplement 2.

### Randomization

Participants were randomly assigned in a 1:1 allocation ratio using computer-generated randomization with permuted blocks of 5 participants. Randomization occurred after the study information was collected. The randomization process was automated through the study website using the R package blockrand, version 1.5 (R Project for Statistical Computing).

### Masking

Participants were masked to treatment allocation through limited disclosure, with the participant information sheet and consent form written so as not to reveal that the study was performed to evaluate a predictive tool. The research team remained masked to treatment allocation throughout the study, including during analysis.

### Outcomes and Measures

The primary outcome was the difference in willingness for surgery between allocation groups at 6 months after enrollment. Willingness for surgery was assessed at multiple time points (baseline, immediately after tool use, 6 weeks, 12 weeks, and 6 months). A binary question, “Are your knee symptoms so bothersome that you would be willing to undergo surgery if medically fit to do so (yes/no),” was used to assess the primary outcome. Furthermore, participants who underwent TKA during the study period were automatically considered willing for surgery at all subsequent follow-up time points. For participants in the TAU group, willingness for surgery at the immediately-after-tool-use time point was considered the same as willingness for surgery at baseline.

Secondary outcomes included treatment preference and decision quality, evaluated using the Knee Decision Quality Instrument (K-DQI) at 6 months only.^23^ Treatment certainty and treatment preference was defined by the participant’s response to question 6 of the K-DQI: “Which treatment do you want to do to treat your knee osteoarthritis?” If participants selected not sure, then they were defined as uncertain with their treatment preference. Furthermore, participant treatment preference was defined by their preferred treatment for knee OA (knee replacement or nonsurgical treatment) as per question 6 of the K-DQI. Demographic information, including age, sex, comorbidities, and body mass index, was captured at baseline, as well as patient-reported outcome measures as determined using the EuroQol 5 Dimensions and Veterans-RAND 12 component score. Race and ethnicity data were not captured as the sample size would have been too low (and ethnicity proportions in the study population too homogenous) to provide any meaningful subanalysis.

### Sample Size

Our initial minimum sample size as published in the study protocol was 169 participants from each study site.^19^ This sample size was calculated to detect an absolute change in willingness for surgery from 65% to 50% based on previous literature.^7^ While we successfully reached the target sample size at the HCF site, we encountered challenges in meeting the target at the SVHM site. The primary reason for this shortfall was a notable decrease in the number of arthroplasty patients at SVHM during the COVID-19 pandemic. Given the persistently low patient numbers throughout the study’s duration, we decided to conclude the study within its originally planned timeline despite not achieving the intended sample size at SVHM.

### Statistical Analysis

The statistical analysis followed the predetermined plan outlined in the study protocol.^19^ Complete case analysis was used to analyze the data. The primary outcome was evaluated using proportions of participants who were willing to undergo surgery at each time point. Statistical tests (χ^2^ test or Fisher exact test for categorical outcomes, 2-tailed *t* test for continuous outcomes, and Wald test for binomial logistic regression) were performed to identify differences between participants in each allocation group. Normality of continuous data was verified using visual inspection of histograms and normal probability plots. Data transformations (square roots, cube roots, and logarithms) were considered for skewed continuous outcome variables. Binomial logistic regression models were used to evaluate treatment effects on willingness for surgery, treatment uncertainty, and preference for surgical treatment. Adjusted logistic regression models were used to account for a difference observed in baseline willingness for surgery (eMethods 3 in Supplement 2). In line with the study protocol, treatment effects are presented as odds ratios (ORs) with 95% CIs or risk differences between groups with respect to willingness for surgery based on predictive margins (mean marginal effect)^24^ (eMethods 2 in Supplement 2). Subanalysis of decile groupings (low, deciles 1-3; medium, deciles 4-6; high, deciles 7-10) and recruitment site were performed to assess the influence of predictive outcomes on treatment effects. However, due to the low proportion of participants who were in the high decile group, subanalysis by decile was not possible for all outcomes. A sensitivity analysis of the primary outcome was performed using a complete dataset developed using multiple imputation methods^25^ (eMethods 1 in Supplement 2). The secondary outcome of decision quality was measured based on the responses to the K-DQI^23^ and evaluated as the proportion of participant responses for each question. The statistical significance threshold was prespecified and set at a 2-sided *P* < .05 for all analyses. Analyses were performed using R, version 4.1.1 and Stata, version 17.0 (StataCorp LLC).

## Results

### Study Population

Of 211 randomized participants, 105 were allocated to the intervention group and 106 were allocated to the TAU group. The intervention and TAU groups showed comparable baseline characteristics (except for baseline willingness), as shown in Table 1. The mean (SD) age of the study cohort was 65.8 (8.3) years, with 118 female participants (55.9%) and 93 male participants (44.1%). There was a higher ratio of participants recruited from HCF compared with SVHM, although the proportion of HCF and SVHM participants in the intervention and TAU groups were comparable. Participants from HCF generally reported better functional status at baseline compared with those from SVHM (eTable 2 in Supplement 2). There was 100% completeness in the capture of baseline participant characteristics and patient-reported outcome measures (Table 1).

**Table 1.** Comparison of Baseline Characteristics and Patient-Reported Outcome Measures

Characteristic	Participants, No. (%)	
	Predictive tool (n = 105)	Treatment as usual (n = 106)
**Demographics**		
Age, mean (SD), y	65.4 (8.43)	66.2 (8.16)
Sex		
Female	53 (50.5)	65 (61.3)
Male	52 (49.5)	41 (38.7)
BMI, mean (SD)	30.2 (6.42)	31.4 (7.26)
Previous knee surgery		
No	66 (62.9)	69 (65.1)
Yes	39 (37.1)	37 (34.9)
Time with knee symptoms, mean (SD), y	7.71 (6.64)	7.70 (6.12)
Smoking status		
Never	58 (55.2)	63 (59.4)
Former	41 (39.0)	41 (38.7)
Current	6 (5.7)	2 (1.9)
**Comorbidities**		
Cardiac		
No	92 (87.6)	93 (87.7)
Yes	13 (12.4)	13 (12.3)
Respiratory		
No	101 (96.2)	103 (97.2)
Yes	4 (3.8)	3 (2.8)
Mental health		
No	96 (91.4)	97 (91.5)
Yes	9 (8.6)	9 (8.5)
Diabetes		
No	95 (90.5)	93 (87.7)
Yes	10 (9.5)	13 (12.3)
**Previous nonsurgical OA treatment** ^a^		
Lifestyle changes		
No	33 (31.4)	21 (19.8)
Yes	72 (68.6)	85 (80.2)
Physical therapy		
No	45 (42.9)	48 (45.3)
Yes	60 (57.1)	58 (54.7)
Nonopioid analgesia		
No	26 (24.8)	20 (18.9)
Yes	79 (75.2)	86 (81.1)
Opioid analgesia		
No	89 (84.8)	88 (83.0)
Yes	16 (15.2)	18 (17.0)
Intra-articular steroid injection		
No	83 (79.0)	81 (76.4)
Yes	22 (21.0)	25 (23.6)
**Recruitment site**		
Private (HCF)	94 (89.5)	94 (88.7)
Public (SVHM)	11 (10.5)	12 (11.3)
**Baseline willingness for surgery**		
Willing	61 (61.0)	74 (69.8)
Not willing	41 (39.0)	32 (30.2)
**EQ5D dimension**		
Mobility		
I have no problems with walking about	23 (21.9)	19 (17.9)
I have some problems with walking about	82 (78.1)	87 (82.1)
I am confined to bed	0	0
Self-care		
I have no problems with self-care	95 (90.5)	91 (85.8)
I have some problems with washing or dressing myself	10 (9.5)	15 (14.2)
I am unable to wash or dress myself	0	0
Usual activities		
I have no problems with performing my usual activities	30 (28.6)	25 (23.6)
I have some problems with performing my usual activities	72 (68.6)	75 (70.8)
I am unable to perform my usual activities	3 (2.9)	6 (5.7)
Pain or discomfort		
I have no pain or discomfort	4 (3.8)	0
I have moderate pain or discomfort	85 (81.0)	83 (78.3)
I have extreme pain or discomfort	16 (15.2)	23 (21.7)
Anxiety or depression		
I am not anxious or depressed	72 (68.6)	75 (70.8)
I am moderately anxious or depressed	33 (31.4)	29 (27.4)
I am extremely anxious or depressed	0	2 (1.9)
**Veterans-RAND 12 component score, mean (SD)**		
Mental	52.5 (10.8)	49.9 (11.1)
Physical	35.5 (7.72)	35.1 (8.23)
Utility	0.72 (0.13)	0.69 (0.14)

Abbreviations: BMI, body mass index (measured as weight in kilograms divided by height in meters squared); EQ5D, EuroQol 5 Dimensions; HCF, Hospitals Contribution Fund; OA, osteoarthritis; SVHM, St Vincent’s Hospital, Melbourne.

^a^ Participants were asked (binary outcome) whether they had ever received or used any of the nonsurgical OA treatments in the past for their knee.

### Trial Implementation

**Figure 1.**
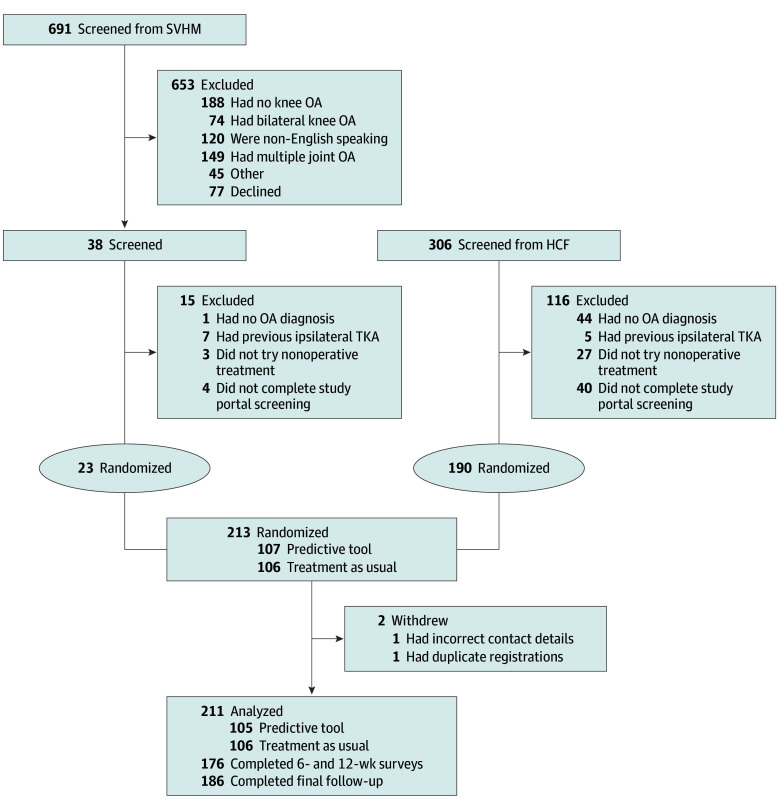
Screening, Allocation, and Follow-Up of the Study Cohort HCF indicates Hospitals Contribution Fund; OA, osteoarthritis; SVHM, St Vincent’s Hospital, Melbourne; TKA, total knee arthroplasty.

In total, we assessed 997 individuals for eligibility, 784 of whom were deemed ineligible due to various exclusion criteria or declined to participate (Figure 1). After the screening and elimination processes, a final count of 213 participants were enrolled into the trial. Two participants were withdrawn at the beginning of the study after randomization (1 for incomplete contact details and 1 for accidental duplicate registration [the original registration was included in the study]), leaving 211 participants included in the analysis. In total, 176 participants (83.4%) completed surveys at 6 weeks and 12 weeks, and 186 (88.2%) completed the final follow-up survey at 6 months.

### Primary Outcome: Willingness for Surgery by Allocation Group

**Figure 2.**
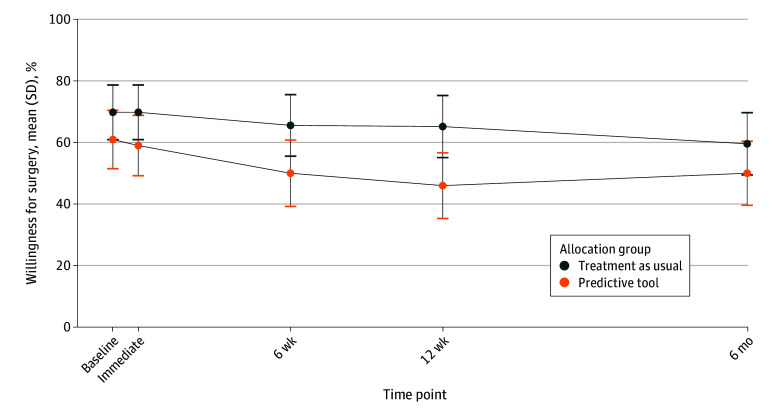
Percentage of All Participants Willing to Undergo Surgery Across All Follow-Up Time Points Analyses of subcohorts are available in eFigures 1 to 3 in Supplement 2.

There was no significant difference in willingness for surgery at 6 months after using the predictive tool. Despite randomization, the mean proportion of participants willing to undergo surgery at baseline was higher in the TAU group (69.8%) compared with the predictive tool group (61.0%) (*P* = .20) (Figure 2; eFigures 1-3 in Supplement 2). For the TAU group, willingness for surgery declined to a mean of 52.8%. Adjusting for baseline differences in willingness for surgery (eTable 1 in Supplement 2), subsequent willingness for surgery was not statistically significant at all follow-up time points (Table 2). The adjusted OR of willingness for surgery at 6 months was 0.85 (95% CI, 0.42-1.71; *P* = .64).

**Table 2.** Adjusted and Unadjusted Treatment Effects of Predictive Tool Use on Willingness for Surgery and Treatment Preference

Outcome measure	OR (95% CI)	*P* value
**Willingness for surgery**		
Unadjusted		
Immediate	0.62 (0.35-1.11)	.10
6 wk	0.53 (0.29-0.96)	.04
12 wk	0.46 (0.25-0.83)	.01
6 mo	0.68 (0.38-1.21)	.19
Adjusted for baseline difference in willingness for surgery		
Immediate	0.70 (0.27-1.81)	.46
6 wk	0.58 (0.26-1.29)	.18
12 wk	0.52 (0.25-1.10)	.09
6 mo	0.85 (0.42-1.71)	.64
**Treatment preference at 6 mo**		
Unadjusted		
Uncertain	0.34 (0.14-0.77)	.01
Prefers surgery^a^	0.43 (0.22-0.84)	.01
Prefers nonsurgical treatment^a^	2.31 (1.19-4.49)	.01
Adjusted for baseline difference in willingness for surgery		
Uncertain	0.36 (0.16-0.76)	.009
Prefers surgery^a^	0.46 (0.20-1.03)	.06
Prefers nonsurgical treatment^a^	2.16 (0.98-4.92)	.06

Abbreviation: OR, odds ratio.

^a^ If not uncertain about treatment.

### Secondary Outcome: Treatment Preference and Decision Quality

The effect of the predictive tool on treatment preference and decision quality (as measured using the K-DQI) are presented in Table 3. In general, tool use did not result in a significant change in decision quality markers. Specific to a single response to the K-DQI, the predictive tool group reported a higher preference for nonsurgical treatment of knee OA at 6 months (48 participants [45.7%]) vs the TAU group (26 participants [24.5%]) (*P* < .001). However, subsequent analysis indicated a nonsignificant treatment effect (adjusted OR, 2.16; 95% CI, 0.98-4.92; *P* = .06) (Table 2).

**Table 3.** Knee Decision Quality Instrument Outcome Measures

Outcome measure	Participants, No. (%)		*P* value
	Treatment as usual (n = 106)	Predictive tool (n = 105)	
“Did any of your health care providers talk about knee replacement surgery as an option for you?”			
Yes	80 (75.5)	75 (71.4)	.52
No	12 (11.3)	16 (15.2)	
Missing	14 (13.2)	14 (13.3)	
“How much did you and your health care providers talk about the reasons to have knee replacement surgery?”			
A lot	26 (24.5)	27 (25.7)	.93
Some	33 (31.1)	34 (32.4)	
A little	26 (24.5)	22 (21.0)	
Not at all	7 (6.6)	8 (7.6)	
Missing	14 (13.2)	14 (13.3)	
“How much did you and your health care providers talk about the reasons not to have knee replacement surgery?”			
A lot	9 (8.5)	15 (14.3)	.60
Some	26 (24.5)	25 (23.8)	
A little	31 (29.2)	27 (25.7)	
Not at all	26 (24.5)	24 (22.9)	
Missing	14 (13.2)	14 (13.3)	
“Did any of your health care providers talk about nonsurgical treatments as something that you should seriously consider?”			
Yes	52 (49.1)	46 (43.8)	.51
No	40 (37.7)	45 (42.9)	
Missing	14 (13.2)	14 (13.3)	
“Which treatment do you think is most likely to provide relief from knee pain caused by osteoarthritis?”			
Surgery	48 (45.3)	58 (55.2)	.10
Nonsurgical treatment	11 (10.4)	13 (12.4)	
About the same	34 (32.1)	20 (19.0)	
Missing	13 (12.3)	14 (13.3)	
“Which treatment do you want to do to treat your knee osteoarthritis?”			
Knee replacement	40 (37.7)	32 (30.5)	<.001
Nonsurgical treatment	26 (24.5)	48 (45.7)	
Not sure	27 (25.5)	11 (10.5)	
Missing	13 (12.3)	14 (13.3)	

## Discussion

This RCT investigated the effect of a novel predictive tool on participant willingness for TKA among individuals with knee OA. The findings revealed that use of the predictive tool did not result in a significant reduction in willingness for surgery within the 6-month follow-up period after adjusting for baseline differences in willingness for surgery. Furthermore, the secondary outcomes of treatment preference and decision quality demonstrated no significant effects of the tool, again after adjusting for baseline differences in willingness for surgery. The study offers key considerations for optimizing the potential benefits of predictive tools in future research, especially with the primary objective of guiding treatment decisions to enhance health outcomes based on patient preferences.

Despite the absence of statistically significant treatment effects on willingness for surgery observed in this study, it is important to recognize that predictive tools might still enhance health outcomes even without a mean change in willingness for surgery. This potential for enhanced health outcomes is particularly relevant because predictive tools could improve patient stratification, especially in scenarios where there are long surgical waiting lists (eg, identifying patients more or less likely to benefit from surgery), without altering the overall willingness for surgery. Ideally, predictive tools should aim to postpone surgery for individuals predicted to have less favorable postoperative outcomes until a more opportune time arises. Consequently, this approach could potentially reduce the proportion of patients undergoing TKA who later express dissatisfaction with the surgical outcome.

To contextualize our study in the landscape of other decision tools, Bansback et al^7^ recently implemented an educational tool among patients with knee OA and reported improvements across multiple domains of the K-DQI after tool use. This divergence from our findings, where only the treatment preference question of the K-DQI (question 6) showed a difference, underscores the multifaceted nature of patient decision quality. The study by Bansback et al^7^ provided a more comprehensive educational package for patients, encompassing various aspects of knee OA and its treatment options, whereas our study primarily focused on predicting the likelihood of improvement after TKA. Furthermore, our intervention was relatively weak as it only provided a surgical prediction at a single time point. This discrepancy in outcomes suggests that patient decision quality may be influenced by not only education about predicted outcomes following surgery but also a holistic understanding of knee OA and its various treatment avenues. With the progressive nature of knee OA symptoms over time, use of a predictive tool across multiple time points may yield more significant results with respect to patient decision-making. Future research should continue to explore the multifaceted components that contribute to informed and high-quality decisions in the context of knee OA management.

One of the major clinical implications of this study is the potential for improved patient selection for TKA. The decision to proceed with TKA is a result of discussions between patient and clinician and not simply a matter of patient preference. However, providing personalized information for patients about their likelihood of success from surgery may help to better inform that discussion or delay it until the patient is more likely to benefit. Ultimately, this information may lead to a reduction in the proportion of dissatisfied patients undergoing TKA, as those with a lower predicted likelihood of improvement may opt for alternative treatment options or delay surgery until it becomes more clinically appropriate.^26^ Based on previous research, this approach is appropriate given that most participants predicted to have a lower likelihood for improvement had earlier or milder symptoms of knee OA.^2,18,27,28,29^

The economic implications of reduced patient preference for TKA due to predictive tool use are also worth considering. Total knee arthroplasty is a costly procedure, and directing health care expenditure toward patients who are more likely to benefit from surgery can lead to a more efficient allocation of resources.^30^ By avoiding unnecessary surgeries in patients with a lower predicted likelihood of improvement, health care systems may experience cost savings while still delivering appropriate care to patients who may be most likely to benefit from TKA.^31,32^

### Limitations

The study encountered several important limitations. First, despite randomization, there was a notable difference in baseline willingness for surgery between participants in the predictive tool and TAU groups. While this difference was not statistically significant, it necessitated adjustments in the analysis to account for this baseline variation. Two main justifications guided these adjustments^33^: (1) the magnitude of the baseline willingness difference was substantial, with an 8.8% advantage favoring the tool, and (2) the primary outcome of willingness for surgery at 6 months is directly linked to baseline willingness for surgery. Given that the purpose of a randomized study is to balance covariates (both measured and unmeasured), the adjustments were deemed appropriate considering the evident imbalance in the data. Future studies may benefit from considering stratified randomization based on baseline willingness to mitigate such disparities.

Second, the decline in willingness for surgery over time observed in the TAU group, from 69.8% at baseline to 52.8% at 6 months, may be attributed to the regression to the mean phenomenon associated with the fluctuating severity of knee OA symptoms.^34^ While this phenomenon is not a direct limitation for this study, as it had both a treatment and control group, it should be considered in quasi-experimental preintervention and postintervention studies, which may be susceptible to regression to the mean.^30^ Additionally, as is being discovered in subsequent qualitative studies in progress, some participants may have misinterpreted the decile scores provided by the predictive tool, leading to an unexpected influence on their willingness for surgery in the opposite direction.

Third, the influence of a predictive tool on health outcomes hinges on its effect on the decision-making process regarding surgery. In our study, the expressed willingness for surgery serves as a proxy for the determination to move forward with the surgical procedure. However, it is essential to note that our study did not explicitly delve into the correlation between this expressed willingness for and the actual occurrence of the surgery. In practical terms, the utility of the tool may be more inclined toward reinforcing an existing decision, be it to proceed with surgery, as observed in the SVHM cohort, or to defer surgery, as noted in the HCF cohort, rather than instigating substantial alterations in decisions related to surgery. While our predictive tool is designed to guide patients away from premature surgery and redirect them to a more opportune time for optimal outcomes, the study does not explicitly measure the effect of such delays on long-term health outcomes.

Fourth, the study’s sample size was smaller than initially projected due to recruitment challenges, particularly at the site where participants were already on a surgical waiting list. This limited our ability to draw statistically significant conclusions about the tool’s effectiveness for those unlikely to benefit but already awaiting surgery. Furthermore, the study’s generalizability may be somewhat constrained, as a larger proportion of participants came from a cohort with access to private health insurance, which typically represents early-stage knee OA. Nonetheless, this cohort is important as it represents one of the largest groups of patients considering TKA in Australia.^35^

## Conclusions

In this RCT, the use of a predictive tool did not reduce willingness for TKA in patients with knee OA up to 6 months after tool use. Furthermore, the predictive tool did not improve patient preference for nonsurgical treatments and did not improve decision quality around TKA. Additional research, including qualitative studies and replication trials, are needed to optimize the design and implementation of predictive tools, address limitations, and fully understand their effect on the decision-making process in TKA.

## Notes

Supplement 1.
Study Protocol

Supplement 2. **eTable 1.** Change in Willingness for Surgery Compared With Baseline, Stratified by Allocation Group and Decile
eMethods 1.

eMethods 2.

eMethods 3.
**eFigure 1.** Histogram of Probability Scores for the Treatment-as-Usual (TAU) Group **eFigure 2.** Histogram of Probability Scores for the Tool Group **eFigure 3.** Histogram of Probability Scores for the Overall (Whole) Cohort **eTable 2.** Differences in Baseline Symptoms and Function by Recruitment Site

Supplement 3.
Data Sharing Statement
