# Supplementary material for: Predictive Tool Use and Willingness for Surgery in Patients With Knee Osteoarthritis: A Randomized Clinical Trial
Source: JAMA Netw Open. 2024 Mar 8;7(3):e240890. doi: 10.1001/jamanetworkopen.2024.0890 (PMC10924247; doi:10.1001/jamanetworkopen.2024.0890)
Supplement: Supplement 1. — Study Protocol [file jamanetwopen-e240890-s001.pdf]

Evaluating Willingness for Surgery Using the SMART Choice Tool to Predict Outcomes after Total Knee Arthroplasty: A Randomised Controlled Trial

“The SMART Choice Trial”

Study Protocol

Version 5.5
Amended 19 July 2022

Confidential

This document is confidential and the property of The University of Melbourne, HCF Research Foundation, and St. Vincent’s Hospital, Melbourne. No part of it may be transmitted, reproduced, published, or used without prior written authorization from the institutions.

Statement of Compliance

This document is a protocol for a clinical research study. The study will be conducted in compliance with all stipulations of this protocol, the conditions of ethics committee approval, the NHMRC National Statement on Ethical Conduct in Human Research (2018) and the Note for Guidance on Good Clinical Practice (CPMP/ICH-135/95).

NB: This tool was previously known as “PROTO-KNEE” and has now been rebranded SMART Choice (Knee) as of 1 February 2022.

ABBREVIATIONS

Abbreviation	Definition
AE	Adverse event
ANZCTR	Australian New Zealand Clinical Trials Registry
CF	Consent form
CI	Co-Investigator
CONSORT	Consolidation Standards of Reporting Trials
CRF	Case report form
EPR	Electronic patient records
GP	General practitioner
HCF	Hospitals Contribution Fund (Health insurance company)
K-DQI	Decision Quality Instrument (Knee Arthritis specific)
MCID	Minimal clinically important difference
MRO	Medical records online
NSAID	Non-steroidal anti-inflammatory drug
OA	Osteoarthritis
PAS	Patient administration systems
PFA	Patellofemoral arthroplasty
PI	Principal Investigator
PIS	Patient information sheet
PROM	Patient reported outcome measure
SAE	Serious adverse event
SAGER	Sex and Gender Equity in Research
SF6D	Short form survey (6 dimensions)
SMART	St. Vincent's Melbourne Arthroplasty Outcomes (Registry)
SPIRIT	Standard Protocol Items: Recommendations for Interventional Trials
SUSAR	Suspected unexpected serious adverse reaction
SVHA	St. Vincent's Hospitals, Australia
SVHM	St. Vincent's Hospital, Melbourne
TAU	Treatment as usual
TKA	Total knee arthroplasty (= total knee replacement)
TRIPOD	Transparent reporting of a multivariate prediction model for prognosis or diagnosis.
UKA	Unicondylar knee arthroplasty
VR-12	Veterans RAND 12 Item Health Survey
WOMAC	Western Ontario and McMaster Universities Osteoarthritis Index

1. INVESTIGATORS AND FACILITIES

1.1. Study Locations

This study will be conducted primarily online with participants recruited from two cohorts:

- Customers of HCF
- Patients at St. Vincent's Hospital, Melbourne (SVHM)

The research team will be based at:

- Department of Surgery
  Faculty of Medical, Dental and Health Sciences
  The University of Melbourne
- Department of Orthopaedic Surgery
  St. Vincent's Hospital, Melbourne.

1.2. Study Management

1.2.1. Principal Investigator

Dr. Chris Schilling
Senior Research Fellow and Health Economist
Chris.schilling@unimelb.edu.au

1.2.2. Project Team

Contact information for research team members are available on request.

Co-investigators:
Dr. Yushy Zhou
A/Prof. Michelle Dowsey
Prof. Peter Choong
Dr. Samantha Bunzli

Project Officer:
Ms. Lauren Patten

Clinical Trials Co-ordinator
Ms. Claire Weeden

1.2.3. Statistician

Dr. Tim Spelman
Senior Biostatistician
Tim@burnet.edu.au

1.3. Sponsor / Funding

This study is funded through a research grant by the HCF Research Foundation (grant number N/A).

2. PROTOCOL SYNOPSIS

Intervention

The *SMART Choice* is a prognostic tool developed to predict patient satisfaction after total knee arthroplasty (TKA). The tool predicts outcome in the form of a likelihood score for satisfaction after TKA. The tool is patient-focused meaning it can be used without the input of clinicians.

Objectives

To evaluate the effect of the *SMART Choice* prognostic tool with regards to patient willingness for surgery in TKA.

Design

Prospective, single-blinded, randomised controlled trial.

Population

People who are suffering from knee OA and considering TKA are eligible for the study. Participants will be recruited from two sources: HCF and SVHM.

Number of Subjects

Based on our sample size calculations, approximately 400 participants will be required for our study. Participants will be randomly allocated 1:1 into two groups: intervention (prognostic tool use) group and treatment as usual (TAU) group.

Outcomes

The primary outcome of the study is willingness for TKA surgery. Secondary outcomes are optimal timing for tool use and accuracy of predicted outcomes. A nested qualitative study will evaluate the clinical utility of the tool from a consumer perspective. An economic analysis will evaluate the cost benefit of the tool.

Follow-up

Participants will be followed up at six weeks, three months, and six months after their initial assessment. Long term follow-up of participants will be investigated as a linked study to the Australian Joint Registry and the St. Vincent's Melbourne Arthroplasty Outcomes (SMART) Registry.

Study Duration

The study duration is estimated to be 21 months to completion from first enrolment.

3. INTRODUCTION AND BACKGROUND

3.1. Terminology

In this study protocol, the term "prognostic tool" refers to the interface (e.g. website or mobile app) that patients interact with to predict outcomes. In contrast, the term "predictive model" refers to the statistical model(s) and/or machine learning algorithm(s) that the prognostic tool uses to calculate predictive outcomes.

3.2. Background

Knee osteoarthritis (OA) is a progressive and debilitating condition for sufferers. Pain and stiffness are common presenting complaints. Without adequate intervention, functional decline and even complete loss of independence can occur.[10] Lifestyle modification, analgesia and physiotherapy comprise the core of non-operative management.[31] In certain situations, intra-articular injections may delay the need for surgery.[23,29] Failing non-operative management, the definitive treatment option for knee OA is TKA.[24]

Based on registry studies, TKA is generally regarded as a successful procedure.[4,11] The risk of adverse event associated with the surgery is relatively low and the probability of improving symptoms is relatively high.[13,20] However, recent studies have reported that up to 20 percent of patients remain unsatisfied after TKA.[1] For these patients, ongoing symptoms from their TKA severely impacts their quality of life.[9,32] With a current trend towards more arthroplasty surgery globally, the social and economic impact of TKA dissatisfaction is a fast growing problem.[21]

To address this issue, solutions need to arise from multiple fronts. Improvement in surgical technique and implant design seem to be the most obvious path forward. However, substantial progress has already been made from pioneers of the past. The trajectory of progress from technique and implant design alone is reaching a plateau.[12,25] An alternative solution would be a completely new treatment for knee OA; a solution that addresses both the symptoms and natural history of the disease. Work is underway to experiment with biologic agents aimed at regenerating cartilage and bone.[15,17,19,28] However, this process is expensive and time consuming without any guarantee of success. Research must therefore explore complementary pathways to find solutions for TKA dissatisfaction.

One of these pathways is through improvement of patient specific factors. The goal here is to optimise patients to become excellent surgical candidates. Prognostic tools fit into this area of research. These are tools developed to predict surgical outcomes. This is clinically useful in two ways. First, if poor outcomes can be predicted before surgery, then patients can be stratified into groups based on risk. For high-risk patients, resources can be set aside to improve modifiable risk factors. This will optimise the patients for surgery. Secondly, prognostic tools can manage patient expectations through informed decision making. A patient who understands their potential outcomes may regress their expectations towards what is realistic for their circumstances. This is based on the understanding that a major driver of dissatisfaction is the imbalance between expected and actual outcomes.[30] The hope is that prognostic tools can better align these two perceptions to improve patient satisfaction and influence patient decision making about surgery for the better.

SMART Choice is a patient focused prognostic tool that predicts outcomes after TKA. The term "patient focused" means that a patient can use the tool by themselves before seeing a clinician. The tool was developed using data from the SMART Registry– an extensive arthroplasty registry with over 10,000 patients and more than 20 years follow up time.[14] *SMART Choice* uses the SMART Registry data to provide patients with a likelihood score for a satisfactory outcome after TKA. This benefits patient care through two pathways; 1) improves informed decision making for the patient, and 2) manages patient expectations in preparation for TKA.

3.3. Rationale and Hypotheses

This study forms part of a wider research project investigating improvement in patient selection for TKA. Prognostic tools fit into this research scope because they can stratify patients based on risk and predicted outcomes. Patients deemed high risk of unsatisfactory outcome can be identified before surgery. Modifiable risk factors can be optimised in these patients to improve outcomes.

Additionally, patients who have knowledge of their predicted outcomes are better informed to make decisions about surgery. Our hypothesis is that prognostic tools in this context can positively influence patient decision making for TKA. We will measure patient decision making through willingness for surgery scales. For patients who are identified as high risk, we hypothesise that a significant proportion of patients will reconsider their willingness for surgery and/or make lifestyle changes to reduce their modifiable risk factors. Furthermore, we expect early intervention with the prognostic tool (before seeing an Orthopaedic Surgeon) to have a larger impact on willingness for surgery than later in the patient journey (after seeing an Orthopaedic Surgeon). The rationale behind this hypothesis is that with progression along the TKA journey, patients will likely have a stronger perception of investment in the process. Patients are therefore less likely to abandon this perceived investment by declining surgery later in the TKA journey.

Although many predictive tools have been developed for TKA and other arthroplasty procedures, very few have been implemented in clinical practice. A possible explanation for this observation is the paucity of clinical trials that have evaluated prognostic tools for arthroplasty. To our knowledge, this will be the first randomised controlled trial (RCT) that will study the effect of prognostic tool use on willingness for surgery in TKA. Willingness for surgery will be used as a proxy measure to understand how prognostic tools can influence patient decision making regarding TKA.

3.4. Research Questions

The overarching research question is "how can we improve patient selection for TKA?" With respect to this randomised controlled trial, the primary question we are investigating is "what are the effects of prognostic tool use on patient willingness for TKA surgery?"

4. STUDY OBJECTIVES

4.1. Primary Objective

The primary objective of this study is to evaluate the impact of the *SMART Choice* tool use on willingness for TKA surgery for patients with knee OA. This is in comparison with standard care alone.

4.2. Secondary Objectives

The secondary objectives of this study are:

- To determine the optimal timing for prognostic tool use in a patient's TKA journey to maximise effect on willingness for surgery.
- To assess the accuracy of *SMART Choice* predicted outcomes compared with actual outcomes in patients who proceeded with surgery (through comparison of patient reported outcome measures).
- To determine if there are differences in the effectiveness of *SMART Choice* when used in sub-populations such as sex, gender, and ethnicity.
- To evaluate the economic benefit of using *SMART Choice*.

Long term (greater than 5 years) follow-up of patients will occur through linkage to the SMART and Australian Joint Registries. This will be registered as a separate study later in the overall project.

5. STUDY DESIGN

5.1. Study Methodology Overview

This is a prospective, assessor-blinded, superiority randomised controlled trial. The trial will be registered with the Australian New Zealand Clinical Trials Registry (ANZCTR). Reporting will be in accordance with the CONSORT Statement.[22] The trial protocol will be published in line with SPIRIT[5] (clinical trial), and SAGER[7] (sex and gender equity) guidelines.

People with knee OA who are considering TKA will be selected for the study. Screening for potential participants will occur from two sources; 1) customers of HCF who are identified as having knee OA and considering TKA, and 2) patients who are referred to SVHM Orthopaedic Outpatients Clinic for consideration of TKA, or on the waiting list for re-review in clinic, or on the waiting list for unilateral TKA. Additional participants outside of these two sources will also be considered, for example, when participants invite friends or family to participate in the study. Participants must be considering TKA surgery and provide informed consent to participate in the study.

Participants will be randomised into two equal groups:

- Intervention (prognostic tool)
- Treatment as usual (TAU)

Definitions for each group are detailed in section 6.

The TAU group is the control group of the study. This represents the care a patient would receive if they were not part of this study and seeking treatment for their knee OA through public channels within the Australian health system.

Due to the nature of the study, only assessor blinding will occur. For the purposes of the study, the research team will be separated into two groups by their roles. There will be research assistants whose primary role is to manage the recruitment and data entry aspects of the study. There will also be investigators who will perform the data analysis of the study. We do not intend for crossover to occur between these roles. Consequently, research assistants will be able to perform their duties unblinded whilst investigators will remain blinded to participant identity and allocation groups during the analysis phase. This blinding strategy prevents bias from being introduced by investigators when interpreting the results of the study.

Blinding of participants would be a challenge logistically. Therefore, the study will instead utilise a limited disclosure method after allocation. Participants will be blinded from what the specific intervention in each arm of the study entails. For surgeons who are reviewing patients in the clinic, they will be blinded to the allocation grouping and have no role in outcome ascertainment. The statistician will be blinded to both arms of the study.

The primary outcome will be the effect of prognostic tool use on participant willingness for TKA surgery. Secondary outcomes will be optimal timing for prognostic tool use, differences in tool effectiveness amongst sub-populations, and accuracy of prognostic tool prediction (in patients who undergo TKA) with respect to satisfactory surgery outcome.

To measure the outcomes of interest, the following data will be collected:

- Willingness for surgery question: "Are your knee symptoms so bothersome that you would be willing to undergo surgery if medically fit to do so? (Yes/No)."[22]
    - If yes, "In what time frame are willing to have surgery?" [Time in months].
- Proceeding with TKA: "Have you already received a TKA for your knee symptoms? (Yes/No)."
- PROM tools:
    - Veterans RAND 12 Item Health Survey Score (VR-12)
    - EQ-5D-3L Questionnaire

PROM data will be captured for the TKA even if the patient has proceeded with TKA during the duration of the study. See section 9.4 and 9.5 for more details.

Participants will be followed up for six months. Data will be captured at four timepoints throughout the study:

- Initial assessment at time of recruitment
- 6 weeks after initial assessment
- 12 weeks after initial assessment
- 6 months after initial assessment

See section 8 for further details about assessments and follow up.

5.2. Number of Subjects

A power analysis calculation was performed to estimate the sample size for this study. We used baseline willingness for surgery in TKA as published by Bendich et al and Dell'Isola et al.[2,8] Methods described in *Fundamentals of Biostatistics 7th Edition* were used for power analysis.[26] Alpha was set at 0.05 and power was set at 80%. From the two comparison studies, a sample size range of 240-360 participants were needed for the study. Additional studies were used to benchmark our sample size calculations to ensure consistency.[6,18] To account for lost to follow up, 5% inflation was included in our final sample size calculation. This buffer is a conservative estimate given the SMART Registry achieves 98% survey follow-up at one year.[14] Our final sample size estimate was 400 participants. The sample size calculated is a feasible number to recruit because SVHM perform approximately 300 TKA each year and HCF currently have approximately 50,000 members with knee OA. We will aim to recruit 200 patients from the SVHM cohort and approach a random pool of all patients with knee OA in the HCF database. Based on these calculations, we estimate that 6 months will be a sufficient time period for recruitment to be completed. In the event that disruptions, including but not limited to COVID-19, negatively affects or delays recruitment, an interim analysis will be performed at 200 participants. The purpose of the interim analysis will be to evaluate the feasibility and necessity of recruitment numbers. See section 7.1 for further details.

5.3. Expected Duration of Study

The study is expected to be completed within 21 months (Fig. 1). This timeline is divided into three phases:

- Phase 1 – study planning, logistics, setting up the appropriate infrastructure to ensure all relevant data can be captured.
- Phase 2 – recruitment of patients will occur sequentially alongside follow up of patients who have been recruited earlier in the study.

- Phase 3 – final follow up of the patients recruited later in the study, data analysis, and preparing the findings for dissemination in journal publications and conferences.

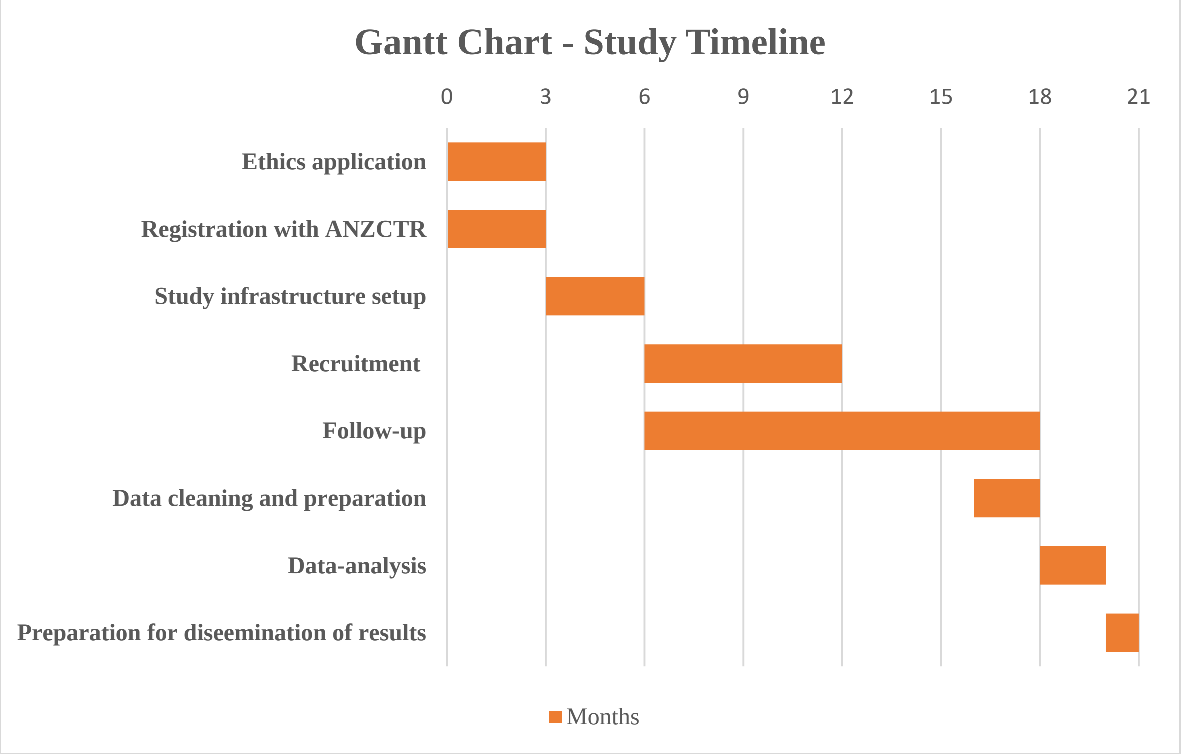

Figure 1. Gantt Chart detailing the timeline of the study.

6. **STUDY TREATMENTS**

6.1.Treatment Arms

To meet the primary and secondary objectives of this study, participants will be randomised into two equal groups.

- **Intervention group** – this group is defined as using the *SMART Choice* tool in addition to standard care for TKA pathway.
- **Treatment as usual (TAU) group** – this group is defined by the absence of prognostic tool use and only receiving usual care for patients on the TKA pathway.

The purpose for randomising participants into intervention and TAU groups is to comparatively measure the outcomes attributed to prognostic tool use. Therefore, the intervention in this study is the use of the prognostic tool (*SMART Choice*). It is important to note that intervention groups receive prognostic tool use *in addition* to standard care. In other words, no care is withheld in the intervention group. By contrast, the TAU group receives no additional intervention from the study.

Sub-analysis will be used to measure secondary outcomes. In particular, optimal timing of prognostic tool use on willingness for surgery will be measured. Participants will be required to indicate their position in the TKA journey at the beginning of the study (see section 10). Time to appointment with an Orthopaedic Surgeon will be used as the reference point. Participants who have not seen an Orthopaedic Surgeon and do not have an appointment to see an Orthopaedic Surgeon for at least 6 weeks are considered early in the TKA journey. Participants who are due to see an Orthopaedic Surgeon within the next 6 weeks are considered in the middle of the TKA journey. Finally, participants who have already seen an Orthopaedic Surgeon are considered late in the TKA journey.

Our hypothesis is that use of the prognostic tool earlier in the TKA patient journey will lead to larger influences on willingness for surgery. The major reasoning behind this hypothesis is because the participants who are later in the TKA journey may feel more invested to continue with the process and undergo TKA despite risks of unsatisfactory outcome.

6.2.Predictive Model Development

The SMART Registry will be used as the primary database to build the predictive model. A combination of literature review and clinical judgement will form the basis for predictor selection. All predictors are required to be patient-specific – meaning the patient can input these variables without clinician knowledge such as radiographic assessment of disease. Univariate and multivariate regression analysis will provide statistical evaluation of correlation between predictors and outcomes. Multiple predictive models using regression modelling and machine learning algorithms will be constructed and compared. 10-fold cross validation technique will be used to internally validate the model thereby reducing bias and overfitting of data. The outcome of interest for the model will be utility score improvement based on SF6D. Minimal clinically important difference (MCID) will be used to assess the threshold of PROM score improvement as satisfactory outcome. A decision curve analysis may be performed to assess the clinical utility of the final model. Furthermore, alternate models investigating hip arthroplasty may also be developed for comparison. The model

development process will be reported using TRIPOD guidelines; Transparent reporting of a multivariate prediction model for prognosis or diagnosis.

The predictive model will be housed as a patient-focused prognostic tool on two user platforms – website for computer and website optimised for mobile/tablet devices. The prognostic tool can be used once by participants in the intervention group. For participants in the TAU group, the prognostic tool will not be available for use until after the study ends. At the conclusion of the study, the prognostic tool will be available for all participants to use freely.

6.2.1 Crossover and Contamination Policy

For the duration of the study, the *SMART Choice* tool will not be live and will only be able to be accessed through direct links to a secure platform. This will prevent patients in the TAU group from accessing the tool during the study and causing crossover contamination.

Alternative prognostic tools for TKA are already available freely on the internet.[16] However, with limited disclosure – the study will be framed as a self-assessment and education intervention. This will prevent participants in the TAU group from actively seeking out alternative prognostic tools for use. If a participant in the TAU uses a prognostic tool during the study, they will not be excluded. This reflects the pragmatic nature of the study because alternative prognostic tools are already freely available for general use.

7. SUBJECT ENROLLMENT AND RANDOMISATION

7.1.Recruitment

Participants will be recruited from two sources:

- HCF client database who have identified patients with knee OA and considering TKA
- SVHM Orthopaedic Outpatient Clinic patients who have presented, been booked, or referred for new appointments to discuss TKA; or are on the waitlist for re-review in clinic, or are on the surgical waitlist for TKA.

NB: Additional participants recruited outside of these sources will be also considered in circumstances such as when participants refer friends or family to the study. For these participants, ethical oversight will be held by the University of Melbourne Ethics Committee.

Based on our sample size calculation, we will need approximately 400 patients in total for the study to achieve our primary objective. As already described in section 5, we will approach 200 patients in the SVHM source and a random pool of all HCF members with knee OA considering TKA. This strategy was deemed the most feasible when balancing the need to recruit enough participants within a reasonable allocated timeframe. As a result, there may be an over-recruitment of participants, from the HCF source.

From the HCF source, potential participants will be recruited from two major outlets. The first will be a news article published in the HCF internal magazine overviewing our trial. The article will provide potential participants to contact us to express their interest in participating in the trial. The second will be the advertisement of the trial recruitment on the HCF HealthShare platform. This is an internal HCF platform where customers can access medical information, including information about how to participate in our trial. From HealthShare, participants can contact us to express their interest in participating in the trial. All participants from HCF who have expressed their interest will be emailed a link to our study website for participation in the trial.

For the SVHM source, potential participants will be selected from monthly Orthopaedic Outpatient Clinic lists for new appointments. On the day of their clinic, research staff will approach potential participants before or after their appointment to consent for the study. An iPad will be available to perform the initial screening and baseline information for the study. In the event that there is an over-recruitment from the HCF cohort, the number of participants recruited from SVHM can be reduced to meet our total sample size calculation. In addition, patients who have been are awaiting FSA, or have been reviewed and are awaiting further review, or have been placed on the surgical waitlist for TKA will be approached for recruitment. Potential participants will receive a phone call from a member of the study team to discuss the study and collect contact details including email to store on a spreadsheet housed on SVHM servers. Participants will be emailed a copy of the PICF and will be asked to respond with “I agree” or similar, to indicate they have read the PICF and agree to participate in the study. Subsequently, the participant will be sent a username and password to access the study website. Please note that for patients who access the study website from the SVHM cohort, no identifiable information will be captured by the website. Instead, a study identifier number will be provided that will match the patient contact details housed on the SVHM spreadsheet.

If the participant is allocated to the intervention group, they will be directed to undertake a baseline questionnaire, willingness for surgery assessment, and PROM scores. Immediately after, they will be asked to use the prognostic tool. The next assessment will then be in 6 weeks' time.

If the patient is allocated to the TAU group, they will be directed to undertake a baseline questionnaire, willingness for surgery assessment, and PROM scores. The next assessment will also be in 6 weeks' time. Section 10 details the workflow of the study in further detail.

The consent form will describe the purpose of the study, the procedures to be followed, and the risks and benefits of participation. A research team member will be available via an on-call research phone (the contact number will be provided) to discuss the study and consent process in further detail.

In the event a potential participant declines our invitation for the study, this will be documented on a recruitment spreadsheet. The patient will be asked to provide a reason for their decline, however, providing this information will be on a voluntary basis. The proportion of patients who decline invitation to the study will be reported in the final study analysis. Furthermore, in the event a participant starts the study but does not complete this, we will contact the participant via email or phone to clarify the intentions of the participant.

The recruitment phase of the study will end on an agreed date as determined by the research team. This will be dependent on the final sample size required. We will perform an interim analysis of the study when we recruit half of the intended sample size. As the sample size calculation was an approximation based on previous results, the sample size range may be as low as 240 (total) up to 400 (total). If the interim analysis demonstrates statistical significance in sample size, then recruitment will cease. If the interim analysis does not demonstrate statistical significance, then the trial will aim for the maximum sample size of 400 (total) but will communicate a firm end date for recruitment. The purpose for documenting a firm end date for recruitment is to prevent ongoing replacement of participants in the event of high lost to follow-up cases. For further details, see Section 7.5.3.

7.2. Eligibility Criteria

7.2.1. Inclusion Criteria

Inclusion criteria will select for the following participants:

- Diagnosed with knee OA and are considering primary unilateral TKA
- Have already trialled non-operative management for their knee symptoms (see Section 5.1)
- Are willing and able to use web or mobile phone based prognostic tool interfaces
- Are able to provide informed consent to participate and available to be followed up for the duration of the study

7.2.2. Exclusion Criteria

Exclusion criteria will select out the following participants:

- Source of knee symptoms is considered to be from any cause other than knee OA e.g. rheumatoid arthritis, hip osteoarthritis, referred lower back pain etc.

- Are considering bilateral TKA, revision TKA, unicondylar knee arthroplasty (UKA), or patellofemoral arthroplasty (PFA)
- TKA on the contralateral side
- Significant bilateral knee symptoms
- Patients younger than 45 years

7.2.3. Eligibility Screening

Participants will be screened for eligibility based on the above criteria. The screening questionnaire will contain the following questions:

- Have you been diagnosed with knee OA?
    - If no, patient will be excluded.
- Which knee is most affected by your symptoms?
    - Left or right.
- Have you already had a knee replacement on your affected knee?
    - If yes, patient will be excluded.
- Have you trialled non-operative treatment for your affected knee, such as pain relief medication, lifestyle changes, or physiotherapy **within the last 12 months**?
    - If no, patient will be excluded.

Other minor eligibility criteria such as previous septic arthritis will be noted on the PIS.

7.3.Randomisation Procedures

Participants will be randomly assigned to receive the prognostic tool use in addition to standard care, or TAU. Simple randomisation of individuals will occur. Equal numbers of participants will be allocated to each group of the study. The allocation process will be built into the research platform that will be provided to potential participants during recruitment (see section 10).

7.4.Study Blinding

Due to the nature of the study, participants will remain unblinded from their allocation group. However, limited disclosure of allocation groups will be applied to prevent participants who are allocated TAU from pro-actively seeking out online prognostic tools to use. Participants will be followed up using the automated software, REDCap (Vanderbilt University, Nashville, TN, USA), which can conceal allocation groups of the participants to the investigators. Follow up queries from participants that require direct communication will be addressed at first instance by a research assistant.

Investigators (excluding research assistants who will not be involved in data analysis) will remain blinded to the allocation group and identity of patients until after final data analysis is performed. Surgeons will be blinded to the allocation group of their patients and will have not influence on the outcome of allocation or intervention.

7.5.Subject Withdrawal

7.5.1. Reasons for Withdrawal

The investigator may withdraw a patient from the study treatment and follow-up procedures if the participant:

- Experiences a serious or intolerable adverse event that may impact their ability to participate in the study independently
- Develops, during the course of the study, symptoms or conditions listed in the exclusion criteria
- Is in the TAU group and uses any prognostic tool for TKA during the course of the study
- Requires early discontinuation for any reason

The investigators will also withdraw all participants from the study treatment if the study is terminated. Participants are free to withdraw from the study at any time upon their request or the request of their legally acceptable representative.

7.5.2. Handling of Withdrawals and Losses to Follow-up

If a participant withdraws from the study, the reasons for withdrawal shall be recorded on a recruitment spreadsheet. Whenever possible, data already captured about that participant will be retained for analysis. Participants who do not formally withdraw from the study but fail to respond to study assessments will be contacted by the research team to redirect compliance with the protocol. This will consist of two documented phone calls and a letter/email follow up. If these contact attempts are unsuccessful, the participant will be deemed lost to follow-up.

7.5.3. Replacements

Participants who have been lost to follow-up or discontinued from the study may be replaced up until the agreed end date for recruitment. This date will be set at least 3 months prior to the end date as agreed by the research team. Lost to follow-up cases that occur after the recruitment phase cannot be replaced and will be noted in the final analysis of data.

7.6.Trial Closures

A participant is considered to have completed the trial if they have completed all phases of the trial including the last assessment as shown in the Schedule of Assessments (see section 8).

8. STUDY VISITS, PROCEDURES AND ASSESSMENTS SCHEDULE

8.1 Schedule of Assessments (SoA)

	SCHEDULE OF ASSESSMENTS						
	Enrolment	Allocation to intervention	Post-allocation				Close-out
TIME POINT*	t_x	t_0	t_1	t_2	t_3	t_4	t_y
ENROLMENT:							
Eligibility screen	X						
Informed consent	X						
Allocation to intervention		X					
ALLOCATION GROUPS:							
Intervention group (I)		X					
Treatment as usual group (TAU)		X					
INTERVENTION:							
Prognostic Tool Use			I				
ASSESSMENTS:							
Baseline questionnaire**		X					
Willingness for surgery^		X		X	X	X	X
Already proceeded with surgery^^				X	X	X	X
PROMs^^^		X		X	X	X	X
Qualitative questionnaires #						X	X

8.2 Definitions for SoA

Terms	Definitions	
* Time Point	t_x	Time at initial contact via email with PIS, CF, and eligibiility screen. If a patient is eligible and consents, allocation will immediately follow this time.
	t_0	Time at allocation with initial concurrent assessments.
	t_1	Immediately after allocation, for prognostic tool use in TAU group.
	t_2	6 weeks after initial assessment (t_0)
	t_3	12 weeks after initial assessment (t_0)
	t_4	6 months after initial assessment (t_0)
	t_y	Time at study closure for individual participant. This should be equal to t_4 if all assessments are completed on schedule.
** Baseline Questionnaire	Questionnaire for all patients who have been allocated in the study. Same for all groups. Captures basic demographic data as well as questions about previous knee treatment and surgery.	
^ Wilingness for Surgery	This assessment asks the question "Are your knee symptoms so bothersome that you wish to undergo surgery if medically fit to do so?" Yes / No. If yes, "In what time frame are you willing to have surgery?" [Time in months].	
^^ Already Proceeded with Surgery	This assessment asks the question "Have you already received a TKA for your knee symptoms?" Yes / No. The purpose for this assessment is to 1) check if the patient has undergone TKA, and 2) outcome aligns with their willingness for surgery response.	
^^^ PROMs	This assessment consists of the VR12 and EQ5D3L questionnaires.	
# Qualitative Questionnaires	This assessment consists of K-DQI (decision quality) and SURE (decisional conflict) tools.	

9. CLINICAL ASSESMENTS

9.1. *Eligibility Screening*

Eligibility screening will be performed prior to allocation using the eligibility criteria described in Section 7.2.3. The screening questionnaire will be electronic and automated. Further eligibility information will be ascertained during discussion prior to informed consent (see Section 9.2).

9.2. *Patient Information Sheet and Consent Form*

Appendix 1 – PICF.

9.3. *Baseline Questionnaire*

The baseline questionnaire provides investigators an understanding of the participant's general characteristics. This questionnaire will only be asked at the first assessment visit during the study (see section 8). The questionnaire would include asking about contact details, date of birth, gender, height, weight, co-morbidities, medications (analgesia), smoking status, previous non-operative management of affect knee OA symptoms, consultation with Orthopaedic Surgeon in the past, appointment to see Orthopaedic Surgeon arranged, previous surgery, previous injury to affected knee, and presence of contralateral TKA.

The questionnaire will be structured to capture the following information:

- Current height / weight
- Current medical conditions
- Smoking status – current, ex-smoker, never
- Question: Have you ever seen an Orthopaedic Surgeon for your affected knee?
    - Yes / No
        - If yes, how long ago did you see your surgeon? (time in weeks)
        - If yes, have you been booked for a knee replacement surgery? (Yes / No)
    - If no, have you been referred to see an Orthopaedic Surgeon for your affected knee?
        - Yes / No
            - If yes, approximately when will you expect to see the Orthopaedic Surgeon? (time in weeks, or "don't know")
- Question: Have you received non-surgical treatment for your affected knee? (Check box)
    - Lifestyle change advice e.g. weight loss, exercise, nutrition
    - Simple analgesia (excluding opioids) e.g. paracetamol, non-steroidal anti-inflammatories (NSAIDs)
    - Opioid analgesia e.g. codeine, tramadol, morphine, oxycodone
    - Physiotherapy
    - Intra-articular injection (steroid)
    - Intra-articular injection (non-steroid) e.g. autologous proteins solution, Synvisc
    - Other

    - None of the above (grey out this option if any of the above are selected)
- Question: Have you ever had surgery before on your affected knee?
    - Yes / No
        - If yes, provide details (free text)

9.4. Regular Assessments

Regular assessments will be conducted in accordance to the SoA. This will consist of “willingness for surgery”, “already proceeded with surgery”, and PROM assessments. Patients will be followed up through automated communication via REDCap software. The timeline on the SoA details the timing for the assessments (see section 8).

9.4.1. *Willingness for Surgery*

This assessment is in the form of a single binary question: “Are your knee symptoms so bothersome that you would be willing to undergo surgery if medically fit to do so? (Yes/No)” If yes, “In what time frame are you willing to have surgery?” [Time in months]. The purpose for this assessment is to evaluate if the patient is still of the mindset to proceed with surgery. This is used as a proxy measurement in place of actual procession with surgery, as the latter would require substantially longer follow up beyond the scope of this study.

9.4.2. *Already Proceeded with Surgery*

This assessment asks the single binary question: “Have you already received a TKA for your knee symptoms? (Yes/No).” The purpose of this assessment is to 1) correlate true outcomes for willingness for surgery if applicable, and 2) differentiate PROM data between knee OA symptoms (if not proceeded with surgery yet) or TKA outcomes. This will not be asked at the initial assessment.

9.4.3. *PROMs*

For this study, we will use patient EQ-5D-3L (Appendix 6) and VR-12 (Appendix 3) as the PROM tools. The justification for using these specific tools is due to the SMART Registry already capturing this data from patients. Given the development of the SMART CHOICE tool will come from SMART Registry data, we will be able to better align our outcomes from this study to the prognostic tool predictions. Participants will be asked to complete all PROMs at each regular assessment schedule.

9.5. Wellbeing Check

All participants will be offered the opportunity to provide feedback or raise concerns about their wellbeing at any point for the duration of the study. This opportunity will also be reminded to participants at each regular assessment timepoint.

10. **STUDY WORKFLOW**

This workflow describes the stepwise process for how the study will be conducted.

10.1. Initial Screening

10.1.1 HCF Source

As described in 7.1, HCF will advertise this study on two platforms; their internal magazine and their HealthShare platform. The internal magazine will use an article about knee replacements to advertise the study. Patients will follow instructions on these platforms to contact us to express interest in the study. No explicit screening will occur from this source, except the screening questions as part of the website (section 7.2.3). The HealthShare platform will ask potential participants a single initial screening question: *"Do you suffer from knee arthritis and are considering a knee replacement?"*

10.1.2 SVHM Source

Access to prospective and retrospective Orthopaedic Outpatient clinic appointments will be screened for patients who:

- Have been booked a new appointment for consideration of unilateral primary knee replacement, and/or
- Have been referred by their General Practitioner (GP) for unilateral knee pain, likely OA
- Have recently (within 12 months) had a consultation with an Orthopaedic Surgeon to consider unilateral primary knee replacement AND not yet undergone surgery
- Awaiting re-review with an Orthopaedic Surgeon for knee surgery
- On the surgical waiting list awaiting total knee replacement

This information will be gathered from two sources:

- Clinic lists available on the SVHM patient administrative system
- Query with SVHM administrative staff who are responsible for booking clinic appointments

From this pool of patients who have been identified as potentially suitable for the study, a research assistant will check their electronic patient records (EPR; at SVHM the systems used are PAS and MRO) for major exclusion criteria such as:

- History of rheumatoid arthritis
- Significant bilateral knee symptoms (through old clinic letters or referral notes)
- Previous contralateral TKA
- Under 45 years old

All patients who pass the initial screening process will be contacted for eligibility screening and formal recruitment (up until 200 patients have been formally recruited into the study or the recruitment phase ends, whichever comes first).

10.2. Eligibility Screening and Formal Recruitment

For participants in the SVHM cohort who pass the initial screening, a brief phone call will be made to inform them of the study before a subsequent email is sent with a link to the study website. For participants in the HCF cohort who pass the initial screening, the link to the study website will be available on the advertising platform(s).

If the participant clicks on the link, they will be directed to a secure online form where they can conduct the study. They will provided with a copy of the PICF and asked to acknowledge before proceeding. Potential participants will be asked to answer the eligibility screening questions, as described in section 7.2.3:

- Have you been diagnosed with knee OA?
    - If no, patient will be excluded.
- Which knee is most affected by your symptoms?
    - Left or right.
- Have you already had a knee replacement on your affected knee?
    - If yes, patient will be excluded.
- Have you trialled non-operative treatment for your affected knee, such as pain relief medication, lifestyle changes, or physiotherapy **within the last 12 months**?
    - If no, patient will be excluded.

If any of the responses result in the participant being ineligible for the study, the participant will be notified they have been excluded and thanked for their time. Participants will be encouraged to contact the research team if they have any questions about their eligibility.

If the participant passes the eligibility screen, they will continue the study through the website.

At any point during this process, the participant will have the ability to stop and not proceed with the recruitment process by exiting the browser. Participants who have registered with the study but not completed the first stage will be recorded. Researchers can then contact the participants to remind them to complete the study,or ask if they would like to be excluded from the study.

10.3. Initial Assessment

All participants who have been formally recruited to the study will then continue with the online forms to undergo the initial assessment. This will comprise of:

- Baseline questionnaire (section 9.3)
    - Current height / weight
    - Current medical conditions
    - Current medications (including over the counter medications such as pain relief)
    - Smoking status – current, ex-smoker, never
    - Question: Have you ever seen an Orthopaedic Surgeon for your affected knee?
        - Yes / No
            - If yes, how long ago did you see your surgeon? (time in weeks)
            - If yes, have you been booked for a knee replacement surgery? (Yes / No)
        - If no, have you been referred to see an Orthopaedic Surgeon for your affected knee?
            - Yes / No
                - If yes, approximately when will you expect to see the Orthopaedic Surgeon? (time in weeks, or “don’t know”)

- Question: Have you received non-surgical treatment for your affected knee? (Check box)
  - Lifestyle change advice e.g. weight loss, exercise, nutrition
  - Simple analgesia (excluding opioids) e.g. paracetamol, non-steroidal anti-inflammatories (NSAIDs)
  - Opioid analgesia e.g. codeine, tramadol, morphine, oxycodone
  - Physiotherapy
  - Intra-articular injection (steroid)
  - Intra-articular injection (non-steroid) e.g. autologous proteins solution, Synvisc
  - Other
  - None of the above (grey out this option if any of the above are selected)
- Question: Have you ever had surgery before on your affected knee?
  - Yes / No
    - If yes, provide details (free text)

- Willingness for surgery (section 9.4.1)
  - "Are your knee symptoms so bothersome that you would be willing to undergo surgery if medically fit to do so? (Yes/No)" If yes, "In what time frame are you willing to have surgery?" [Time in months].
- PROM scores (section 9.4.3)
  - VR-12
  - EQ-5D-3L

After the initial assessment is complete, the participant will be allocated automatically through the browser (and randomly via computer random number generator). The participant will be blinded to their allocation arm.

10.4. Allocation

Allocations will be recorded on REDCap, but limited disclosure will be used in this study to conceal the intervention allocated to the participant. Participants in the intervention group will be presented the results of the *SMART Choice* tool. A notice will thank the participant for their contribution to the study, and will remind them that their next assessment will arrive via email in 6 week's time.

If a participant is allocated to the TAU group, they will receive a notice that thanks them for their participation in the study so far, and similarly will be reminded that their next assessment will arrive via email in 6 week's time. Although participants in the TAU group will not have the results of *SMART Choice* presented to them, their probability scores will be recorded discretely for comparison to the intervention group.

10.5. Regular Assessments

Regular assessments will occur via email link to the participant as per the SoA. This will automated using the REDCap software. There will be a single version of REDCap housed on University of Melbourne servers for HCF and SVHM patients. REDCap will send email follow up to assess willingness for surgery, already proceeded with surgery, and PROMs at 6 weeks, 12 weeks, and 6 months after initial assessment. The format for assessing these metrics will be similar to the initial assessment. All regular assessments will provide an opportunity for participants to feedback on the study and their wellbeing.

10.6. Data Collection

All data will be collected online as the participant enters their information. Data will be stored on secure servers at either HCF or SVHM. See detail data management plan in section 13 for further details.

10.7. Trial Closure

After the 6-month regular assessment, the participant will be notified that the study has concluded for them. Participants in the TAU group will be provided a standalone link to use the *SMART Choice* tool if they wish. Contact details will once again be provided to participants should they have any queries or concerns after conclusion of the study.

11. NESTED QUALITATIVE STUDY

11.1. Synopsis

A nested qualitative study will be incorporated into the main clinical trial. The purpose of this study is to evaluate the user experience of *SMART Choice* for patients. Specifically, experiences regarding patient decision conflict and knowledge will be assessed. Qualitative analysis will be obtained from two methods: 1) qualitative questionnaires, and 2) semi-structured interviews.

The former method will utilise an exploratory standpoint to investigate constructs that play a role in our main trial outcomes. On the contrary, the latter method will be used to confirm our *a priori* assumptions and hypotheses about what constructs might play a role in our main trial outcomes. Both methods are needed to provide a holistic understanding of the user experience for *SMART Choice*.

11.2. Qualitative Questionnaires

All participants in the main clinical trial will be sent two additional questionnaires via email at the time of their final assessment. These questionnaires are validated from previous research to produce discriminatory and reproducible results.[3,27] The addition of these questionnaires will provide a cross-sectional understanding of how useful the *SMART Choice* tool was for patient decision-making.

The first questionnaire is the "Knee Osteoarthritis Decision Quality Instrument" (K-DQI). This questionnaire is specific for patients who suffer from knee osteoarthritis. The questionnaire aims to assess:

- Which aspects of decision-making matter most to the patient
- How well the patient is understanding the information provided, and
- The level of communication between patient and clinician prior to decision-making.

Appendix 4 – K-DQI.

The second questionnaire is a short screening tool to assess decisional conflict. It consists of four binary items using the acronym "SURE".

SURE Acronym	Items	Yes [1]	No [0]
Sure of myself	Do you feel SURE about the best choice for you?	☐	☐
Understand information	Do you know the benefits and risks of each option?	☐	☐
Risk-benefit ratio	Are you clear about which benefits and risks matter most to you?	☐	☐
Encouragement	Do you have enough support and advice to make a choice?	☐	☐

If a patient answers "no" to 1 or more questions, then the screen is considered positive for decisional conflict. Understanding decisional conflict is important to ensure the information provided by *SMART Choice* is presented with clarity and aids the overall experience for patients on their TKA journey.

Appendix 5 – SURE.

11.3. Semi-Structured Interviews

Participants from the intervention group will be asked to take part in semi-structured interviews. Purposive sampling will be used to ensure equal representation of participants (e.g. gender and age range) from each study cohort (HCF and SVHM) will be recruited. The recruitment process will be ongoing until saturation of common themes is met. As such, there is no sample size calculation possible and this is standard practice for qualitative study methodology.

Participants must provide additional consent to take part in the semi-structured interviews. This will be included as an optional section to the PICF for the main clinical trial.

The aims of the interviews are to:
- Explore the barriers and enablers of *SMART Choice* adoption into clinical practice
- Understand the processes underlying the main trial outcomes

With these aims, the interviews ask the following overarching research questions:
- What are the participant's perceptions of the *SMART Choice* tool?
- In what ways did the *SMART Choice* tool impact on the decision-making process?
- Why are patients willing or unwilling to undergo TKA after using the *SMART Choice* tool?

Example questions that will be asked in the interviews are:
- Can you describe the *SMART Choice* tool to me?
- How easy or hard did you find the tool to use?
- How do you think we could make it easier for people to use in the future?
- Can you explain to me how you think TKA could help you? Why do you think this?
- The *SMART Choice* tool indicated your likely outcome from TKA is X – what do you think about this?
- You have indicated you are (un)willing to undergo TKA surgery – why is this?

The interviews will be conducted by an experienced and trained qualitative researcher. Each interview will take approximately 1 hour to complete. The setting of the interview can be face-to-face (subject to COVID-19 restrictions) or over telephone / video call. The interview contents will be audio recorded and transcribed. Thematic analysis will be used to group common themes obtained from the interviews.

11.3.1. Data Storage of Semi-Structured Interviews

All interviews will be audio recorded with the audio file securely stored and separated from transcripts. Verbal consent will be recorded as part of the interview. Transcription will be performed by a transcription company that is associated with and complies with The University of Melbourne's research integrity and privacy policies. All transcripts will be de-identified. Audio recordings will be destroyed at the conclusion of the study. Transcripts will be stored for a further 7 years, then destroyed. The data management plan outline for the semi-structured interviews are in addition to the data management plan for the project (section 13).

12. ECONOMIC ANALYSIS OF SMART CHOICE

12.1. Synopsis

An economic analysis of the *SMART Choice* tool will be conducted in line with standard economic principles. De-identified participant data will be assigned with cost utilities that have been standardised. The outcome will be to calculate the cost-benefit and economic impact of using the *SMART Choice* tool in the context of willingness for surgery, and reduction in non-beneficial TKAs.

13. DATA MANAGEMENT PLAN

13.1. Data Collection

All data collection will follow guidelines as mandated by Australian Privacy laws. Data will be collected from the online forms used for regular assessments and *SMART Choice* tool website.

13.2. Data Storage

Initial attempts were made to separate HCF and SVHM data collection via REDCap with UniMelb and SVHM servers, respectively. The reason for this was to retain SVHM patient information on SVHM servers. However, the SVHM REDCap system does not have the capacity to send email follow up surveys whereas the UniMelb REDCap system does. This is a critical function of REDCap that is required for the study to progress to completion. As a result, all participants in the study will have their study information uploaded to UniMelb REDCap to ensure that follow up questionnaires are emailed at our specified follow up timepoints. All data that is housed on UniMelb REDCap servers can only be accessed by study team members that are affiliated with UniMelb, have a UniMelb login and password, and are able to pass 2-factor authentication. Specific to SVHM, the only identifiable information that will be housed on UniMelb REDCap servers is the first name, last name, email address, and mobile phone number.

For participants who use the SMART Choice website, data will be captured and stored on a secure server at The University of Melbourne. This will only be accessible by University staff who are directly involved in this project (named on this protocol) and have a unique username and password. For participants from the SVHM source, this information will be de-identified and given a study ID instead. For participants from other sources, identifiable information will be captured and stored.

13.3. General Principals

All participants in the intervention arm of the study will have access to the *SMART Choice* tool website via their own unique login credentials. These credentials will entitle the participant to access a limited version of the website. User account permissions will be limited so participants can only see their *SMART Choice* responses. Administrator account permissions will be available only to investigators and research assistants who can create new user accounts, see/search for participants, and export datasets.

Furthermore, the *SMART Choice* tool website will not be accessible by public search engines. This limits accessibility of the tool to only participants with a link and login credentials. Once the study concludes, a version of the website that hosts the *SMART Choice* tool as a standalone format will be open access.

All soft copy patient identifiable documents will be located on a password protected computers in restricted access spaces. For participants in the SVHM cohort, information will be stored within SVHM computer networks physically located at SVHM. For remote access, two-factor authentication is required. For participants in the HCF cohort, information will be stored within The University of Melbourne computer networks. Similary, remote access to

this network also requires two-factor authentication. Study dataset codebooks will be password protected.

All hard copy patient identifiable documents will be in a restricted office area on SVHM location. All consent forms will be stored separately to other project information.

13.4. Study Record Retention

All data collected will be stored for a minimum of 7 years after the conclusion of the study.

14. ADMINISTRATIVE ASPECTS

14.1. Confidentiality

Subject confidentiality is strictly held in trust by the participating investigators, research staff, and the study institution(s). The study protocol, documentation, data and all other information generated will be held in strict confidence. No information concerning the study or the data will be released to any unauthorized third party, without prior written approval of the sponsoring institutions (HCF, SVHM, and The University of Melbourne). Authorized representatives of the sponsoring institution may inspect all documents and records required to be maintained by the PI, limited to their recruited participants, including but not limited to, medical records (office, clinic or hospital) and pharmacy records for the subjects in this study. The clinical study site will permit access to such records. Clinical information will not be released without written permission of the subject, except as necessary for monitoring by Ethics Committee or regulatory agencies.

14.2. Independent Ethics Committee Approval

This protocol and the informed consent document and any subsequent modifications will be reviewed and approved by the Ethics Committees of The University of Melbourne and SVHA. A letter of ethics approval by both Committees will be obtained prior to the commencement of the study, as well as approval for other study documents subject to the Committees reviews.

14.3. Modifications of the Protocol

This study will be conducted in compliance with the current version of the protocol. Any change to the protocol document, PIS, or CF that affects the scientific intent, study design, patient safety, or may affect a participants willingness to continue participation in the study is considered an amendment, and therefore will be written and filed as an amendment to this protocol and/or CF. All such amendments will be submitted to the local Ethics Committees, for approval prior to becoming effective.

14.4. Protocol Deviations

All protocol deviations must be recorded in the patient record (source document) and on a CRF and must be reported to the PI. Protocol deviations will be assessed for significance by the PI. Those deviations deemed to have a potential impact on the integrity of the study results, patient safety or the ethical acceptability of the trial will be reported to the local Ethics Committee within 14 days.

Where deviations to the protocol identify issues for protocol review, the protocol will be amended as per section 13.3

14.5. Participant Reimbursement

Participants will not be reimbursed for the main clinical trial. However, for participants in the semi-structured interviews a $25AUD supermarket voucher will be offered for their time.

13.6. Financial Disclosure and Conflicts of Interest

All investigators and research staff must declare any conflicts of interest or financial interest relating to the study prior to involvement with the study. This will be disclosed in accordance with The University of Melbourne and SVHA Research Integrity Policies.

REFERENCES

1. Baker PN, van der Meulen JH, Lewsey J, Gregg PJ, National Joint Registry for England and Wales. The role of pain and function in determining patient satisfaction after total knee replacement. Data from the National Joint Registry for England and Wales. J. Bone Joint Surg. Br. 2007 Jul;89(7):893–900.

2. Bendich I, Halvorson RT, Ward D, Nevitt M. Predictors of a change in patient willingness to have Total knee arthroplasty: Insights from the osteoarthritis initiative. Knee. 2020 Jun;27(3):667–675.

3. Boland L, Légaré F, McIsaac DI, Graham ID, Taljaard M, Dècary S, et al. SURE Test Accuracy for Decisional Conflict Screening among Parents Making Decisions for Their Child. Med. Decis. Making. 2019 Nov;39(8):1010–1018.

4. Carr AJ, Robertsson O, Graves S, Price AJ, Arden NK, Judge A, et al. Knee replacement. Lancet. 2012 Apr 7;379(9823):1331–1340.

5. Chan A-W, Tetzlaff JM, Gøtzsche PC, Altman DG, Mann H, Berlin JA, et al. SPIRIT 2013 explanation and elaboration: guidance for protocols of clinical trials. BMJ [Internet]. 2013 Jan 9 [cited 2021 Oct 11];346. Available from: https://www.bmj.com/content/346/bmj.e7586doi:10.1136/bmj.e7586

6. Chow S-C, Shao J, Wang H, Lokhnygina Y. Sample size calculations in clinical research: Third edition. 3rd ed. Third edition. | Boca Raton : Taylor & Francis, 2017. | Series: Chapman & Hall/CRC biostatistics series | "A CRC title, part of the Taylor & Francis imprint, a member of the Taylor & Francis Group, the academic division of T&F Informa plc.": Chapman and Hall/CRC; 2017.

7. De Castro P, Heidari S, Babor TF. Sex And Gender Equity in Research (SAGER): reporting guidelines as a framework of innovation for an equitable approach to gender medicine. Commentary. Ann. Ist. Super. Sanita. 2016 Apr;52(2):154–157.

8. Dell'Isola A, Jönsson T, Rolfson O, Cronström A, Englund M, Dahlberg L. Willingness to Undergo Joint Surgery Following a First-Line Intervention for Osteoarthritis: Data From the Better Management of People With Osteoarthritis Register. Arthritis Care Res. . 2021 Jun;73(6):818–827.

9. Dhurve K, Scholes C, El-Tawil S, Shaikh A, Weng LK, Levin K, et al. Multifactorial analysis of dissatisfaction after primary total knee replacement. Knee. 2017 Aug;24(4):856–862.

10. Felson DT, Zhang Y, Hannan MT, Naimark A, Weissman BN, Aliabadi P, et al. The incidence and natural history of knee osteoarthritis in the elderly. The Framingham Osteoarthritis Study. Arthritis Rheum. 1995 Oct;38(10):1500–1505.

11. Gandhi R, Tsvetkov D, Davey JR, Mahomed NN. Survival and clinical function of cemented and uncemented prostheses in total knee replacement: a meta-analysis. J. Bone Joint Surg. Br. 2009 Jul;91(7):889–895.

12. Gill GS, Joshi AB, Mills DM. Paper #5 20 year survivorship analysis of total condylar knee arthroplasty. J. Arthroplasty. 1999 Feb;14(2):245.

13. Gøthesen O, Espehaug B, Havelin L, Petursson G, Lygre S, Ellison P, et al. Survival rates and causes of revision in cemented primary total knee replacement: a report from the Norwegian Arthroplasty Register 1994-2009. Bone Joint J. 2013 May;95-B(5):636–642.

14. Gould D, Thuraisingam S, Shadbolt C, Knight J, Young J, Schilling C, et al. Cohort profile: the St Vincent’s Melbourne Arthroplasty Outcomes (SMART) Registry, a pragmatic prospective database defining outcomes in total hip and knee replacement patients. BMJ Open. 2021 Jan 22;11(1):e040408.

15. Gray PC, Choe S. Design-augmented (DA) biologics: BMP chimeras for bone and cartilage regeneration. Osteoarthritis Cartilage. 2020 Feb;28(2):123–125.

16. Gutacker N, Street A. Use of large-scale HRQoL datasets to generate individualised predictions and inform patients about the likely benefit of surgery. Qual. Life Res. 2017 Sep;26(9):2497–2505.

17. Hadley CJ, Shi WJ, Murphy H, Tjoumakaris FP, Salvo JP, Freedman KB. The Clinical Evidence Behind Biologic Therapies Promoted at Annual Orthopaedic Meetings: A Systematic Review. Arthroscopy. 2019 Jan;35(1):251–259.

18. Hawker GA, Conner-Spady BL, Bohm E, Dunbar MJ, Jones CA, Ravi B, et al. The relationship between patient-reported readiness for total knee arthroplasty and likelihood of a good outcome at one year. Arthritis Care Res. [Internet]. 2021 Jan 18;(acr.24562). Available from: https://onlinelibrary.wiley.com/doi/10.1002/acr.24562doi:10.1002/acr.24562

19. Knapik DM, Evuarherhe A, Frank RM, Steinwachs M, Rodeo S, Mumme M, et al. Nonoperative and Operative Soft-Tissue and Cartilage Regeneration and Orthopaedic Biologics of the Knee: An Orthoregeneration Network (ON) Foundation Review. Arthroscopy. 2021 Aug 1;37(8):2704–2721.

20. Liddle AD, Judge A, Pandit H, Murray DW. Adverse outcomes after total and unicompartmental knee replacement in 101 330 matched patients: a study of data from the National Joint Registry for England and Wales. Lancet. 2014 Oct 18;384(9952):1437–1445.

21. Mayfield CK, Haglin JM, Levine B, Della Valle C, Lieberman JR, Heckmann N. Medicare Reimbursement for Hip and Knee Arthroplasty From 2000 to 2019: An Unsustainable Trend. J. Arthroplasty. 2020 May;35(5):1174–1178.

22. Moher D, Hopewell S, Schulz KF, Montori V, Gøtzsche PC, Devereaux PJ, et al. CONSORT 2010 Explanation and Elaboration: updated guidelines for reporting parallel group randomised trials. BMJ [Internet]. 2010 Mar 24 [cited 2021 Oct 11];340. Available from: https://www.bmj.com/content/340/bmj.c869doi:10.1136/bmj.c869

23. Ong KL, Runa M, Lau E, Altman R. Is Intra-Articular Injection of Synvisc Associated with a Delay to Knee Arthroplasty in Patients with Knee Osteoarthritis? Cartilage. 2019 Oct;10(4):423–431.

24. Richmond J, Hunter D, Irrgang J, Jones AMH, Snyder-Mackler L, Daniel Van Durme MD, et al. The treatment of osteoarthritis (OA) of the knee. J. Bone Joint Surg. Am. 2010;92:990–993.

25. Rodriguez JA, Bhende H, Ranawat CS. Total condylar knee replacement: a 20-year followup study. Clin. Orthop. Relat. Res. 2001 Jul;(388):10–17.

26. Rosner B. Fundamentals of Biostatistics (The 7th edition). Boston, MA: Brooks/Cole. 2011;

27. Sepucha KR, Stacey D, Clay CF, Chang Y, Cosenza C, Dervin G, et al. Decision quality instrument for treatment of hip and knee osteoarthritis: a psychometric evaluation. BMC Musculoskelet. Disord. 2011 Jul 5;12:149.

28. Simon TM, Jackson DW. Articular Cartilage: Injury Pathways and Treatment Options. Sports Med. Arthrosc. 2018 Mar;26(1):31–39.

29. Tang A, Almetwali O, Zak SG, Bernstein JA, Schwarzkopf R, Aggarwal VK. Do preoperative intra-articular corticosteroid and hyaluronic acid injections affect time to total joint arthroplasty? J Clin Orthop Trauma. 2021 May;16:49–57.

30. Thompson AG, Suñol R. Expectations as determinants of patient satisfaction: concepts, theory and evidence. Int. J. Qual. Health Care. 1995 Jun;7(2):127–141.

31. Vaishya R, Pariyo GB, Agarwal AK, Vijay V. Non-operative management of osteoarthritis of the knee joint. Journal of Clinical Orthopaedics and Trauma. 2016 Jul 1;7(3):170–176.

32. Verhaar J. Patient satisfaction after total knee replacement—still a challenge. Acta Orthop. 2020 May 3;91(3):241–242.
