# Supplementary material for: Predictive Tool Use and Willingness for Surgery in Patients With Knee Osteoarthritis: A Randomized Clinical Trial
Source: JAMA Netw Open. 2024 Mar 8;7(3):e240890. doi: 10.1001/jamanetworkopen.2024.0890 (PMC10924247; doi:10.1001/jamanetworkopen.2024.0890)
Supplement: Supplement 2. — eTable 1. Change in Willingness for Surgery Compared With Baseline, Stratified by Allocation Group and Decile eMethods 1. eMethods 2. eMethods 3. eFigure 1. Histogram of Probability Scores for the Treatment-as-Usual (TAU) Group eFigure 2. Histogram of Probability Scores for the Tool Group eFigure 3. Histogram of Probability Scores for the Overall (Whole) Cohort eTable 2. Differences in Baseline Symptoms and Function by Recruitment Site [file jamanetwopen-e240890-s002.pdf]

Supplemental Online Content

Zhou Y, Patten L, Spelman T, et al. Predictive tool use and surgery willingness in patients with knee osteoarthritis. *JAMA Netw Open*. 2024;7(3):e240890. doi:10.1001/jamanetworkopen.2024.0890

eTable 1. Change in Willingness for Surgery Compared With Baseline, Stratified by Allocation Group and Decile

eMethods 1.

eMethods 2.

eMethods 3.

eFigure 1. Histogram of Probability Scores for the Treatment-as-Usual (TAU) Group

eFigure 2. Histogram of Probability Scores for the Tool Group

eFigure 3. Histogram of Probability Scores for the Overall (Whole) Cohort

eTable 2. Differences in Baseline Symptoms and Function by Recruitment Site

This supplemental material has been provided by the authors to give readers additional information about their work.

© 2024 Zhou Y et al. *JAMA Network Open.*

eTable 1. Change in Willingness for Surgery Compared With Baseline, Stratified by Allocation Group and Decile

Decile Group	TAU	Predictive Tool	TAU	Predictive Tool	TAU	Predictive Tool
	1-3 (Low Probability for Improvement)		4-6 (Medium Probability for Improvement)		7-10 (High Probability for Improvement)	
Count	74	79	19	18	13	8
Willingness Immediate Post-tool/enrolment* (%)						
Less Willing	NA	9 (11.4)	NA	1 (5.6)	NA	0 (0.0)
More Willing	NA	6 (7.6)	NA	3 (16.7)	NA	0 (0.0)
Unchanged	NA	59 (74.7)	NA	14 (77.8)	NA	8 (100.0)
Missing	NA	5 (6.3)	NA	0 (0.0)	NA	0 (0.0)
Willingness 6 Weeks Post-tool/enrolment (%)						
Less Willing	7 (9.5)	10 (12.7)	2 (10.5)	0 (0.0)	1 (7.7)	0 (0.0)
More Willing	6 (8.1)	2 (2.5)	0 (0.0)	1 (5.6)	0 (0.0)	0 (0.0)
Unchanged	50 (67.6)	51 (64.6)	13 (68.4)	14 (77.8)	11 (84.6)	8 (100.0)
Missing	11 (14.9)	16 (20.3)	4 (21.1)	3 (16.7)	1 (7.7)	0 (0.0)
Willingness 12 Weeks Post-tool/enrolment (%)						
Less Willing	7 (9.5)	14 (17.7)	2 (10.5)	1 (5.6)	1 (7.7)	1 (12.5)
More Willing	4 (5.4)	5 (6.3)	0 (0.0)	1 (5.6)	0 (0.0)	0 (0.0)
Unchanged	50 (67.6)	46 (58.2)	14 (73.7)	13 (72.2)	11 (84.6)	6 (75.0)
NA	13 (17.6)	14 (17.7)	3 (15.8)	3 (16.7)	1 (7.7)	1 (12.5)
Willingness 6 Months Post-tool/enrolment (%)						
Less Willing	12 (16.2)	13 (16.5)	2 (10.5)	2 (11.1)	0 (0.0)	0 (0.0)
More Willing	4 (5.4)	6 (7.6)	0 (0.0)	1 (5.6)	0 (0.0)	0 (0.0)
Unchanged	50 (67.6)	48 (60.8)	14 (73.7)	14 (77.8)	12 (92.3)	8 (100.0)

© 2024 Zhou Y et al. *JAMA Network Open.*

Missing	8 (10.8)	12 (15.2)	3 (15.8)	1 (5.6)	1 (7.7)	0 (0.0)

*TAU: treatment as usual; *participants in TAU group did not use the tool and therefore no immediate post-tool willingness for surgery was recorded.*

NB: Fisher's exact test was used to compare proportions of willingness for surgery at each timepoint. No statistical differences were found.

© 2024 Zhou Y et al. *JAMA Network Open.*

eMethods 1

This supplementary document provides sensitivity analyses of the primary and secondary outcomes – willingness for surgery and treatment preference. The treatment effects are presented in an identical format to the main results of the study, using odds ratios (OR) with 95% confidence intervals.

Sensitivity Analysis 1 – HCF Cohort Only

In this analysis, only participants recruited from the HCF (private health insurance) site are included. There was insufficient sample size of participants from the SVHM (St. Vincent's Hospital Melbourne) site to perform an equivalent sensitivity analysis in this cohort.

Table. Odds ratios for willingness for surgery and treatment preference (unadjusted and adjusted) for HCF cohort only.

Table. Treatment effects of predictive tool use on various outcome measures (HCF Only)

Outcome Measure	Outcome	Adjustment	Timepoint	Odds Ratios	Lower 95%CI	Upper 95%CI	P Value
Willingness for Surgery	Willing for surgery	Unadjusted	Immediate	0.63	0.35	1.14	0.141
			6 Weeks	0.54	0.28	1.01	0.058
			12 Weeks	0.47	0.25	0.89	0.021
			6 Months	0.68	0.36	1.25	0.236
	Willing for surgery	Adjusted for baseline difference in willingness for surgery	Immediate	0.71	0.27	1.84	0.603
			6 Weeks	0.58	0.25	1.13	0.214
			12 Weeks	0.55	0.25	1.17	0.128

© 2024 Zhou Y et al. *JAMA Network Open.*

			6 Months	0.84	0.40	1.76	0.893
Treatment Preference	Feels uncertain about treatment preference	Unadjusted	6 Months	0.33	0.14	0.70	0.005
	Prefers surgical treatment (if not uncertain about treatment)		6 Months	0.45	0.21	0.92	0.031
	Prefers non-surgical treatment (if not uncertain about treatment)		6 Months	2.23	1.08	4.67	0.031
Treatment Preference	Feels uncertain about treatment preference	Adjusted for baseline difference in willingness for surgery	6 Months	0.35	0.15	0.76	0.041
	Prefers surgical treatment (if not uncertain about treatment)		6 Months	0.48	0.20	1.13	0.098
	Prefers non-surgical treatment (if not uncertain about treatment)		6 Months	2.09	0.89	5.06	0.098

Sensitivity Analysis 2 – Imputed Dataset

In this analysis, the “mice” package in R was used to generate an imputed dataset. The multiple imputation process used age, sex, decile, site, allocation group, and willingness at all other timepoints as variables in the model. Chained equations were used to iteratively impute missing values for each variable while considering the observed values of others. We used predictive mean matching as the imputation method.

© 2024 Zhou Y et al. *JAMA Network Open.*

Table. Odds ratios for willingness for surgery and treatment preference (unadjusted and adjusted) for the imputed dataset.

Outcome Measure	Outcome	Adjustment	Timepoint	Odds Ratios	Lower 95%CI	Upper 95%CI	P Value
Willingness for Surgery	Willing for surgery	Unadjusted	Immediate	0.64	0.36	1.13	0.123
			6 Weeks	0.57	0.32	1.01	0.053
			12 Weeks	0.51	0.28	0.91	0.023
			6 Months	0.70	0.40	1.24	0.220
	Willing for surgery	Adjusted for baseline difference in willingness for surgery	Immediate	0.69	0.27	1.78	0.438
			6 Weeks	0.58	0.27	1.25	0.163
			12 Weeks	0.51	0.25	1.04	0.064
			6 Months	0.81	0.41	1.63	0.561
Treatment Preference	Feels uncertain about treatment preference	Unadjusted	6 Months	0.48	0.24	0.93	0.034
	Prefers surgical treatment (if not uncertain about treatment)		6 Months	0.51	0.27	0.94	0.032
	Prefers non-surgical treatment (if not uncertain about treatment)		6 Months	1.98	1.07	3.70	0.032
Treatment Preference	Feels uncertain about treatment preference	Adjusted for baseline difference in willingness for surgery	6 Months	0.49	0.24	0.97	0.044
	Prefers surgical treatment (if not uncertain about treatment)		6 Months	0.58	0.27	1.20	0.153

© 2024 Zhou Y et al. *JAMA Network Open.*

	Prefers non-surgical treatment (if not uncertain about treatment)	6 Months	1.74	0.83	3.67	0.153

© 2024 Zhou Y et al. *JAMA Network Open.*

eMethods 2

This document provides a supplementary analysis of the randomized clinical trial data. The treatment effects of the tool have been provided as a risk difference. This is calculated using the "margins" command in R, which provides marginal effects summaries of logistic regression models. The specific method used involves deriving the average marginal effects, which are the mean of unit-specific partial derivatives of the regression equation with respect to each variable in the model for each unit in the data. In the context of logistic regression, these average marginal effects can be interpreted as risk differences.

The analyses have been performed for the original study cohort, HCF cohort (sensitivity analysis) and imputed dataset (sensitivity analysis). Both unadjusted and adjusted (for baseline willingness for surgery) analyses have been performed. Description of how the imputed dataset was created can be found in other supplementary documents.

Table. Average marginal effects (risk difference) for willingness for surgery across timepoints. Negative average marginal effects indicate a reduction in willingness for surgery for the tool group.

Cohort	Adjustments in Analysis	Timepoint	Average Marginal Effects	95% CI	P Value
Original study cohort	Unadjusted	Immediate	-0.108	-0.238 - 0.022	0.103
		6 Week	-0.156	-0.299 - -0.011	0.035
		12 Week	-0.192	-0.336 - -0.048	0.009

© 2024 Zhou Y et al. *JAMA Network Open.*

		6 Month	-0.096	-0.238 - 0.047	0.188
Original study cohort	Adjusted for baseline willingness for surgery	Immediate	-0.030	-0.110 - 0.050	0.466
		6 Week	-0.076	-0.188 - 0.036	0.184
		12 Week	-0.106	-0.228 - 0.016	0.089
		6 Month	-0.028	-0.147 - 0.091	0.645
HCF study site only (sensitivity analysis)	Unadjusted	Immediate	-0.109	-0.250 - 0.032	0.129
		6 Week	0.153	-0.308 - 0.002	0.053
		12 Week	-0.187	-0.342 - -0.032	0.018
		6 Month	-0.098	-0.250 - 0.055	0.209
HCF study site only (sensitivity analysis)	Adjusted for baseline willingness for surgery	Immediate	-0.033	-0.123 - 0.057	0.476
		6 Week	-0.082	-0.206 - 0.042	0.197
		12 Week	-0.105	-0.240 - 0.029	0.124
		6 Month	-0.031	-0.160 - 0.098	0.640
Imputed dataset (sensitivity analysis)	Unadjusted	Immediate	-0.117	-0.246 - 0.012	0.074
		6 Week	-0.156	-0.287 - -0.025	0.020
		12 Week	-0.137	-0.270 - -0.004	0.044
		6 Month	-0.080	-0.214 - 0.054	0.240
Imputed dataset (sensitivity analysis)	Adjusted for baseline willingness for surgery	Immediate	-0.048	-0.130 - 0.035	0.257
		6 Week	-0.095	-0.194 - 0.005	0.063
		12 Week	-0.084	-0.195 - 0.027	0.137
		6 Month	-0.026	-0.135 - 0.084	0.646

© 2024 Zhou Y et al. *JAMA Network Open.*

eMethods 3

This supplementary document provides a more comprehensive understanding of how the predictive tool generates reports for individuals.

Predictors

The tool generates a logistic regression model based on age, sex, and baseline symptoms (as per the Veterans-RAND 12 responses). Participants in the study input these variables as part of a baseline questionnaire. Both participants in the intervention and control group provide these predictive variables to the study database, but only those in the intervention (tool) group are provided a predictive report.

Table – Co-efficients of variables used in the final logistic regression model.

	Coefficient	Standard Error	Confidence Interval (95%)		P Value
Intercept	0.613	0.037	0.541	0.686	<0.001
Gender	0.13	0.037	0.058	0.203	<0.001
Utility Score Preop	-0.707	0.088	-0.88	-0.537	<0.001
VR12 General Health Preop	-0.416	0.04	-0.495	-0.337	<0.001
VR12 Moderate Activities Preop	-0.002	0.042	-0.083	0.08	0.962
VR12 Emotional Problems Less Preop	-0.147	0.071	-0.286	-0.008	0.038
VR12 Emotional Problems Limit Work Preop	0.06	0.066	-0.069	0.188	0.36
VR12 Pain Interference Preop	0.224	0.051	0.124	0.323	<0.001

© 2024 Zhou Y et al. *JAMA Network Open.*

VR12 Social Activities Preop	0.11	0.053	0.006	0.214	0.039
Age (Category) 64-69	0.046	0.045	-0.044	0.135	0.316
Age (Category) 70-75	-0.047	0.045	-0.135	0.042	0.301
Age (Category) 76-80	-0.077	0.043	-0.162	0.007	0.073
Age (Category) 81+	-0.121	0.04	-0.199	-0.042	0.003

Deciles

The tool generates a probability score 0 (0% likelihood of improvement) to 1 (100% likelihood of improvement) based on the logistic regression model. The probability score is then assigned to a decile based on a range derived from SMART registry hold-out sample.

Table – Probability score ranges associated with each decile.

Decile	Probability for Improvement (median; range)	Sample	Actual Outcome			
			Improvement (n; %)		No Improvement (n; %)	
**1**	0.338 (0.000 - 0.416)	93	30	32.3	63	67.7
**2**	0.476 (0.417 - 0.510)	93	35	37.6	58	62.4
**3**	0.548 (0.511 - 0.576)	93	45	48.4	48	51.6
**4**	0.599 (0.577 - 0.623)	93	59	63.4	34	36.6
**5**	0.642 (0.624 - 0.661)	93	60	64.5	33	35.5

© 2024 Zhou Y et al. *JAMA Network Open.*

**6**	0.684 (0.662 - 0.698)	93	66	71.0	27	29.0
**7**	0.714 (0.699 - 0.735)	92	68	73.9	24	26.1
**8**	0.757 (0.736 - 0.774)	92	70	76.1	22	23.9
**9**	0.795 (0.775 - 0.819)	92	76	82.6	16	17.4
**10**	0.854 (0.820 – 1.000)	92	78	84.8	14	15.2

Distributions

Histogram of probability scores are presented.

© 2024 Zhou Y et al. *JAMA Network Open.*

eFigure 1. Histogram of Probability Scores for the Treatment-as-Usual (TAU) Group

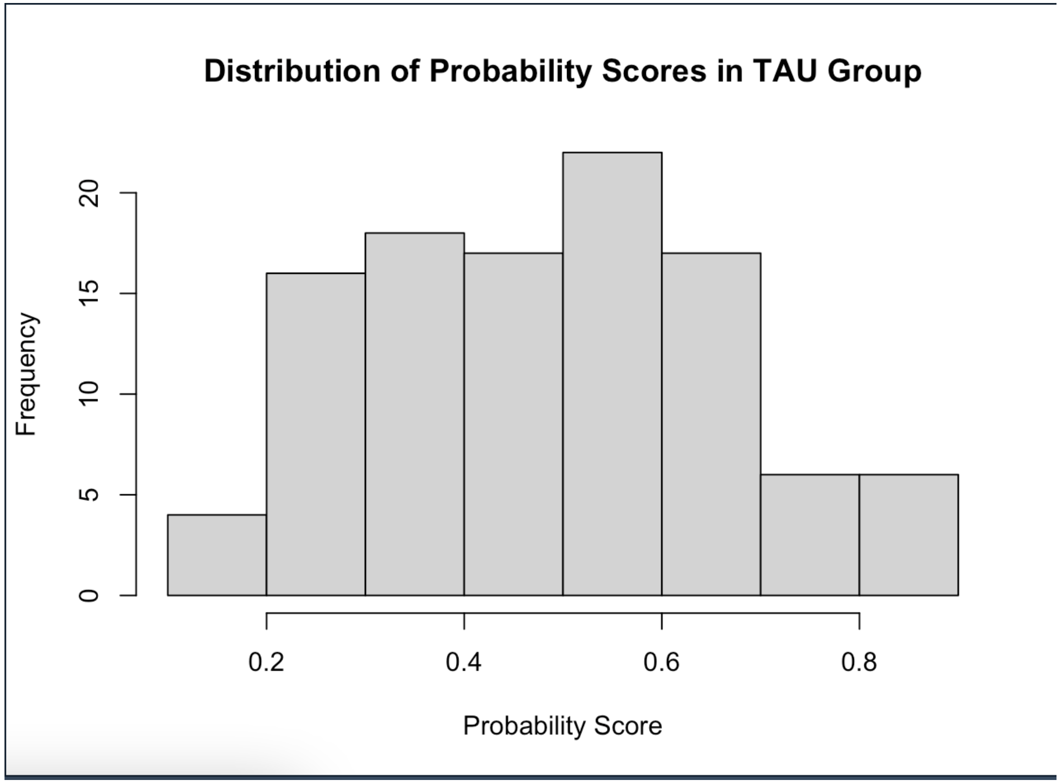

© 2024 Zhou Y et al. *JAMA Network Open.*

eFigure 2. Histogram of Probability Scores for the Tool Group

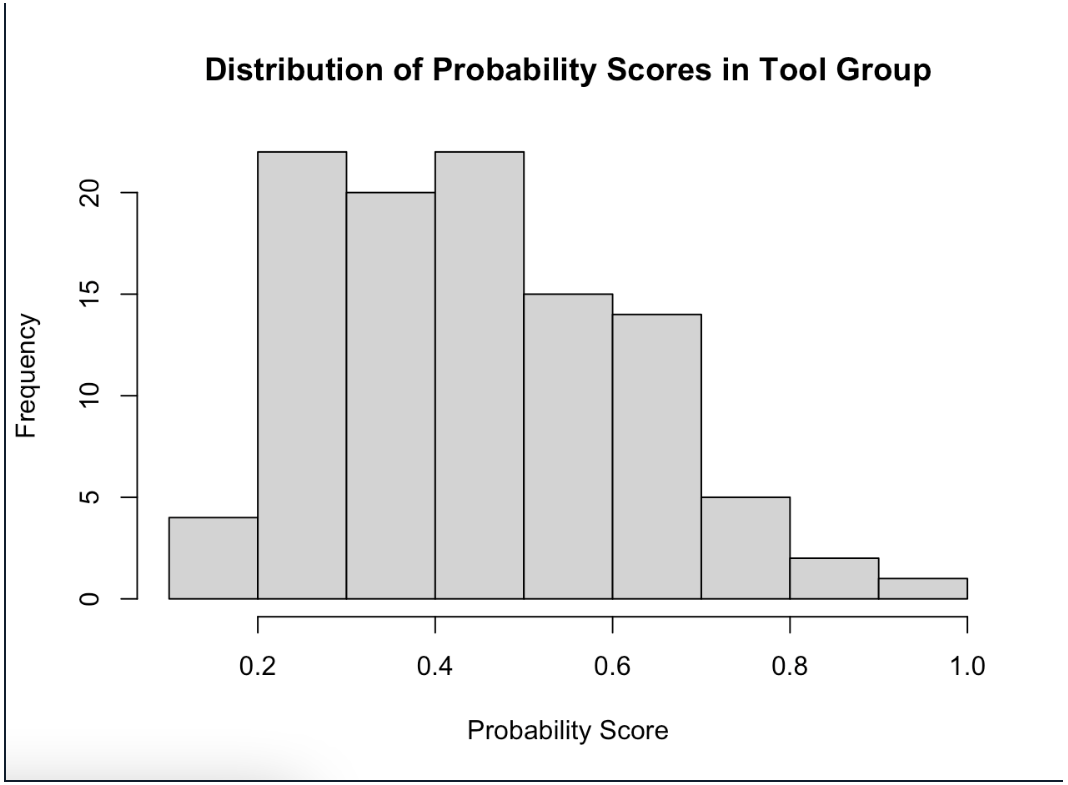

© 2024 Zhou Y et al. *JAMA Network Open.*

eFigure 3. Histogram of Probability Scores for the Overall (Whole) Cohort

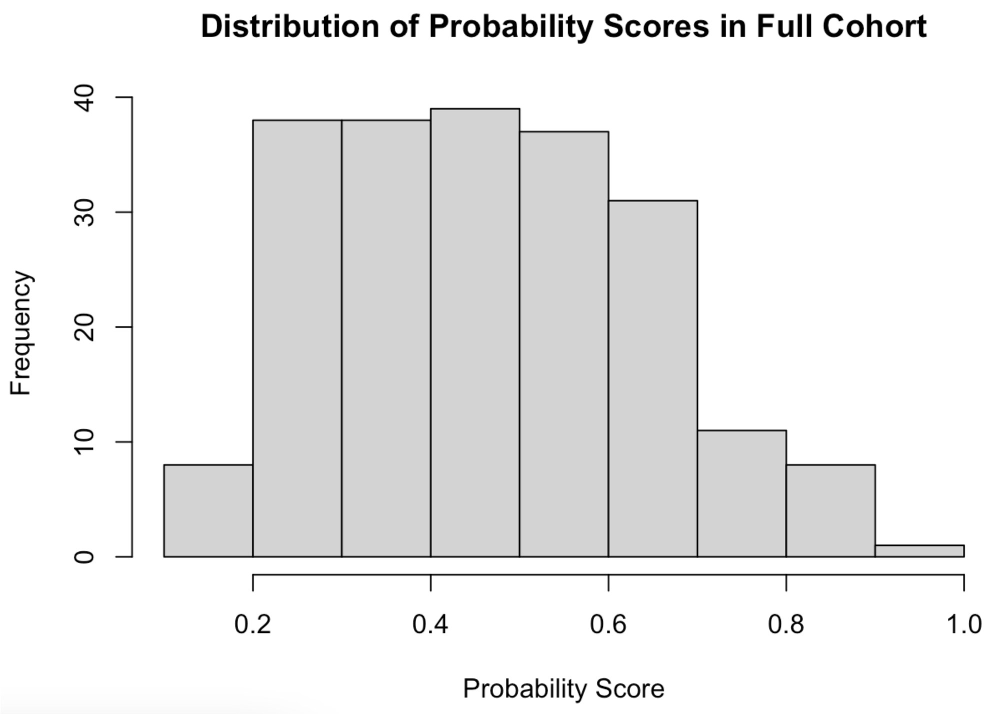

© 2024 Zhou Y et al. *JAMA Network Open.*

Exemplar of the Predictive Tool Questionnaire

Screenshots of the questions used to generate the predictive outcome are attached.

Questionnaire

1. Age

Age

2. Sex

○ Male

○ Female

These questions ask for your views about your health. This information will help keep track of how you feel and how well you are able to do your usual activities. If you are unsure how to answer a question, please give the best answer you can.

3. In general would you say your health is?

○ Poor

○ Fair

○ Good

© 2024 Zhou Y et al. *JAMA Network Open.*

○ Very good

○ Excellent

4. Does your health limit you in **moderate activities,** such as moving a table, pushing a vacuum cleaner, bowling, playing golf? If so, how much?”

○ No, not limited at all

○ Yes, limited a little

○ Yes, limited a lot

During the past 4 weeks, have you had any of the following problems with your work or other regular daily activities **as a result of your physical health?**

5. Such as being limited in the **kind** of work or other activities?

○ No, none of the time

○ Yes, a little of the time

○ Yes, some of the time

○ Yes, most of the time

○ Yes, all of the time

© 2024 Zhou Y et al. *JAMA Network Open.*

Exemplar of the Predictive Outcome Report

Screenshot of the report provided to individuals are attached.

SMART Choice

What is SMART Choice?

The SMART Choice tool has been developed for patients who are considering a knee replacement. The tool uses simple questions about your age, sex and current symptoms to predict your likely outcome after knee replacement. The intention behind developing this tool was to support patients to make better informed decisions about their surgical care.

Results

The SMART Choice Tool has calculated your likely outcome after total knee replacement.

Comparing your results with 100 other patients who are similar to you:

- **85 significantly improved**
- **15 did not improve**

at 12 months after knee replacement surgery.

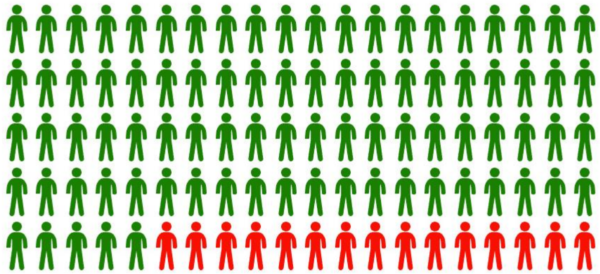

When ranked on your likelihood for improvement after surgery using deciles, **you have been placed in the 10th decile.**

For comparison, patients in the 1st decile have the lowest likelihood for improvement and patients in the 10th decile have the highest likelihood for improvement.

Note: As your circumstances change, your likely outcome after total knee replacement may change as well. Revisiting this tool in future may give you a different result. If you have any questions about your results today and/or total knee replacement, please discuss this with a qualified medical practitioner. For more information about the tool, please see the "Tool" section of the website.

© 2024 Zhou Y et al. *JAMA Network Open.*

eTable 2. Differences in Baseline Symptoms and Function by Recruitment Site

	HCF (Private) (N=188)	SVHM (Public) (N=23)	P-value
**Time with Knee OA Symptoms (Years)**			
Mean (SD)	7.60 (6.36)	8.52 (6.52)	0.52
**Required Non-Opioid Analgesia for Knee OA Symptoms**			
Yes	149 (79.3%)	16 (69.6%)	0.29
No	39 (20.7%)	7 (30.4%)	
**Required Opioid Analgesia for Knee OA Symptoms**			
Yes	22 (11.7%)	12 (52.2%)	<0.001
No	166 (88.3%)	11 (47.8%)	
**Reviewed by Physiotherapist**			
Yes	104 (55.3%)	14 (60.9%)	0.66
No	84 (44.7%)	9 (39.1%)	
**Reviewed by Orthopaedic Surgeon**			
Yes	142 (75.5%)	23 (100%)	0.01
No	46 (24.5%)	0 (0%)	
**Baseline Physical Component Score (VR12)**			
Mean (SD)	36.1 (7.79)	28.8 (6.43)	<0.001
**Baseline Mental Component Score (VR12)**			
Mean (SD)	52.5 (10.2)	40.2 (11.6)	<0.001
**Baseline Utility Score (VR12)**			
Mean (SD)	0.725 (0.126)	0.550 (0.105)	<0.001
**Predicted Outcome (Decile)**			
1-3 (Low likelihood for improvement)	146 (77.7)	7 (30.4%)	<0.001
4-6 (Medium likelihood for improvement)	28 (14.9)	9 (39.1%)	
7-10 (High likelihood for improvement)	14 (7.4)	7 (30.4%)	

HCF: Hospital Contributions Fund; OA: ostearthritis; SD: standard deviation; SVHM: St Vincent's Hospital, Melbourne; VR12: Veterans-Rand 12

© 2024 Zhou Y et al. *JAMA Network Open.*
