# Supplementary material for: Predictive Tool Use and Willingness for Surgery in Patients With Knee Osteoarthritis: A Randomized Clinical Trial
Source: JAMA Netw Open. 2024 Mar 8;7(3):e240890. doi: 10.1001/jamanetworkopen.2024.0890 (PMC10924247; doi:10.1001/jamanetworkopen.2024.0890)
Supplement: Supplement 3. — Data Sharing Statement [file jamanetwopen-e240890-s003.pdf]

Data Sharing Statement

Zhou. Predictive Tool Use and Willingness for Surgery in Patients With Knee Osteoarthritis. *JAMA Netw Open*. Published March 08, 2024. doi:10.1001/jamanetworkopen.2024.0890

Data
Data available: No

Additional Information
Explanation for why data not available: as part of ethical obligations, patient data is not available to be shared.
